# Loci Associated with *N*-Glycosylation of Human Immunoglobulin G Show Pleiotropy with Autoimmune Diseases and Haematological Cancers

**DOI:** 10.1371/journal.pgen.1003225

**Published:** 2013-01-31

**Authors:** Gordan Lauc, Jennifer E. Huffman, Maja Pučić, Lina Zgaga, Barbara Adamczyk, Ana Mužinić, Mislav Novokmet, Ozren Polašek, Olga Gornik, Jasminka Krištić, Toma Keser, Veronique Vitart, Blanca Scheijen, Hae-Won Uh, Mariam Molokhia, Alan Leslie Patrick, Paul McKeigue, Ivana Kolčić, Ivan Krešimir Lukić, Olivia Swann, Frank N. van Leeuwen, L. Renee Ruhaak, Jeanine J. Houwing-Duistermaat, P. Eline Slagboom, Marian Beekman, Anton J. M. de Craen, André M. Deelder, Qiang Zeng, Wei Wang, Nicholas D. Hastie, Ulf Gyllensten, James F. Wilson, Manfred Wuhrer, Alan F. Wright, Pauline M. Rudd, Caroline Hayward, Yurii Aulchenko, Harry Campbell, Igor Rudan

**Affiliations:** 1 Glycobiology Laboratory, Genos, Zagreb, Croatia; 2 Faculty of Pharmacy and Biochemistry, University of Zagreb, Zagreb, Croatia; 3 Institute of Genetics and Molecular Medicine, University of Edinburgh, Edinburgh, United Kingdom; 4 Centre for Population Health Sciences, The University of Edinburgh Medical School, Edinburgh, United Kingdom; 5 Faculty of Medicine, University of Zagreb, Zagreb, Croatia; 6 National Institute for Bioprocessing Research and Training, Dublin-Oxford Glycobiology Laboratory, Dublin, Ireland; 7 Faculty of Medicine, University of Split, Split, Croatia; 8 Radboud University Nijmegen Medical Centre, Nijmegen, The Netherlands; 9 Department of Medical Statistics and Bioinformatics, Leiden University Medical Center, Leiden, The Netherlands; 10 Netherlands Consortium for Healthy Aging, Leiden, The Netherlands; 11 Department of Primary Care and Public Health Sciences, Kings College London, London, United Kingdom; 12 Kavanagh St. Medical Centre, Port of Spain, Trinidad and Tobago; 13 Department of Chemistry, University of California Davis, Davis, California, United States of America; 14 Department of Molecular Epidemiology, Leiden University Medical Center, Leiden, The Netherlands; 15 Department of Gerontology and Geriatrics, Leiden University Medical Center, Leiden, The Netherlands; 16 Biomolecular Mass Spectrometry Unit, Department of Parasitology, Leiden University Medical Center, Leiden, The Netherlands; 17 Chinese PLA General Hospital, Beijing, China; 18 School of Public Health, Capital Medical University, Beijing, China; 19 Graduate University, Chinese Academy of Sciences, Beijing, China; 20 School of Medical Sciences, Edith Cowan University, Perth, Australia; 21 Department of Immunology, Genetics, and Pathology, SciLifeLab Uppsala, Rudbeck Laboratory, Uppsala University, Uppsala, Sweden; 22 Institute of Cytology and Genetics SD RAS, Novosibirsk, Russia; Georgia Institute of Technology, United States of America

## Abstract

Glycosylation of immunoglobulin G (IgG) influences IgG effector function by modulating binding to Fc receptors. To identify genetic loci associated with IgG glycosylation, we quantitated *N*-linked IgG glycans using two approaches. After isolating IgG from human plasma, we performed 77 quantitative measurements of *N*-glycosylation using ultra-performance liquid chromatography (UPLC) in 2,247 individuals from four European discovery populations. In parallel, we measured IgG *N*-glycans using MALDI-TOF mass spectrometry (MS) in a replication cohort of 1,848 Europeans. Meta-analysis of genome-wide association study (GWAS) results identified 9 genome-wide significant loci (P<2.27×10^−9^) in the discovery analysis and two of the same loci (*B4GALT1* and *MGAT3*) in the replication cohort. Four loci contained genes encoding glycosyltransferases (*ST6GAL1*, *B4GALT1*, *FUT8*, and *MGAT3*), while the remaining 5 contained genes that have not been previously implicated in protein glycosylation (*IKZF1*, *IL6ST-ANKRD55*, *ABCF2-SMARCD3*, *SUV420H1*, and *SMARCB1-DERL3*). However, most of them have been strongly associated with autoimmune and inflammatory conditions (e.g., systemic lupus erythematosus, rheumatoid arthritis, ulcerative colitis, Crohn's disease, diabetes type 1, multiple sclerosis, Graves' disease, celiac disease, nodular sclerosis) and/or haematological cancers (acute lymphoblastic leukaemia, Hodgkin lymphoma, and multiple myeloma). Follow-up functional experiments in haplodeficient *Ikzf1* knock-out mice showed the same general pattern of changes in IgG glycosylation as identified in the meta-analysis. As *IKZF1* was associated with multiple IgG *N*-glycan traits, we explored biomarker potential of affected *N*-glycans in 101 cases with SLE and 183 matched controls and demonstrated substantial discriminative power in a ROC-curve analysis (area under the curve = 0.842). Our study shows that it is possible to identify new loci that control glycosylation of a single plasma protein using GWAS. The results may also provide an explanation for the reported pleiotropy and antagonistic effects of loci involved in autoimmune diseases and haematological cancer.

## Author Summary

After analysing glycans attached to human immunoglobulin G in 4,095 individuals, we performed the first genome-wide association study (GWAS) of the glycome of an individual protein. Nine genetic loci were found to associate with glycans with genome-wide significance. Of these, four were enzymes that directly participate in IgG glycosylation, thus the observed associations were biologically founded. The remaining five genetic loci were not previously implicated in protein glycosylation, but the most of them have been reported to be relevant for autoimmune and inflammatory conditions and/or haematological cancers. A particularly interesting gene, *IKZF1* was found to be associated with multiple IgG *N*-glycans. This gene has been implicated in numerous diseases, including systemic lupus erythematosus (SLE). We analysed *N*-glycans in 101 cases with SLE and 183 matched controls and demonstrated their substantial biomarker potential. Our study shows that it is possible to identify new loci that control glycosylation of a single plasma protein using GWAS. Our results may also provide an explanation for opposite effects of some genes in autoimmune diseases and haematological cancer.

## Introduction

**Figure 1:**
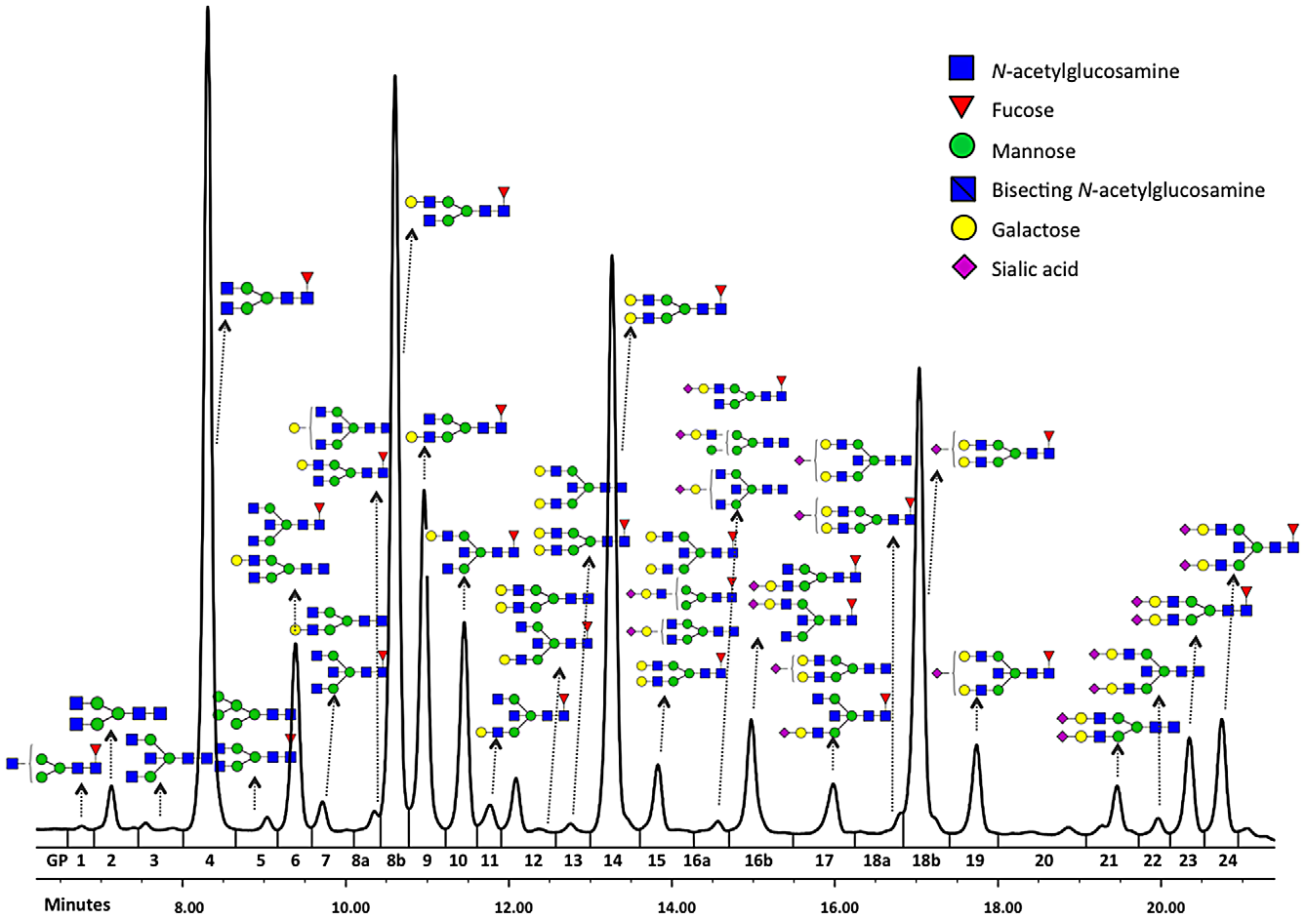
Structures of glycans separated by HILIC-UPLC analysis of the IgG glycome.

Glycosylation is a ubiquitous post-translational protein modification that modulates the structure and function of polypeptide components of glycoproteins [1], [2]. *N*-glycan structures are essential for multicellular life [3]. Mutations in genes involved in modification of glycan antennae are common and can lead to severe or fatal diseases [4]. Variation in protein glycosylation also has physiological significance, with immunoglobulin G (IgG) being a well-documented example. Each heavy chain of IgG carries a single covalently attached bi-antennary *N*-glycan at the highly conserved asparagine 297 residue in each of the CH2 domains of the Fc region of the molecule. The attached oligosaccharides are structurally important for the stability of the antibody and its effector functions [5]. In addition, some 15–20% of normal IgG molecules have complex bi-antennary oligosaccharides in the variable regions of light or heavy chains [6], [7]. 36 different glycans (Figure 1) can be attached to the conserved Asn297 of the IgG heavy chain [8], [9], leading to hundreds of different IgG isomers that can be generated from this single glycosylation site.

Glycosylation of IgG has important regulatory functions. The absence of galactose residues in association with rheumatoid arthritis was reported nearly 30 years ago [10]. The addition of sialic acid dramatically changes the physiological role of IgGs, converting them from pro-inflammatory to anti-inflammatory agents [11], [12]. Addition of fucose to the glycan core interferes with the binding of IgG to FcγRIIIa and greatly diminishes its capacity for antibody dependent cell-mediated cytotoxicity (ADCC) [13], [14]. Structural analysis of the IgG-Fc/FcγRIIIa complex has demonstrated that specific glycans on FcγRIIIa are also essential for this effect of core-fucose [15] and that removal of core fucose from IgG glycans increases clinical efficacy of monoclonal antibodies, enhancing their therapeutic effect through ADCC mediated killing [16]–[18].

New high-throughput technologies, such as high/ultra performance liquid chromatography (HPLC/UPLC), MALDI-TOF mass spectrometry (MS) and capillary electrophoresis (CE), allow us to quantitate *N*-linked glycans from individual human plasma proteins. Recently, we performed the first population-based study to demonstrate physiological variation in IgG glycosylation in three European founder populations [19]. Using UPLC, we showed exceptionally high individual variability in glycosylation of a single protein - human IgG - and substantial heritability of the observed measurements [19]. In parallel, we quantitated IgG *N*-glycans in another European population (Leiden Longevity Study – LLS) by mass spectrometry. In this study, we combined those high-throughput glycomics measurements with high-throughput genomics to perform the first genome wide association (GWA) study of the human IgG *N*-glycome.

## Results

### Genome-wide association study and meta-analysis

We separated a single protein (IgG) from human plasma and quantitated its *N*-linked glycans using two state-of-the-art technologies (UPLC and MALDI-TOF MS). Their comparative advantages in GWA studies were difficult to predict prior to the conducted analyses, so both were used - one in each available cohort. We performed 77 quantitative measurements of IgG *N*-glycosylation using ultra performance liquid chromatography (UPLC) in 2247 individuals from four European discovery populations (CROATIA-Vis, CROATIA-Korcula, ORCADES, NSPHS). In parallel, we measured IgG *N*-glycans using MALDI-TOF mass spectrometry (MS) in 1848 individuals from another European population (Leiden Longevity Study (LLS)). Descriptions of these population cohorts are found in Table S11. Aiming to identify genetic loci involved in IgG glycosylation, we performed a GWA study in both cohorts. Associations at 9 loci reached genome-wide significance (P<2.27×10^−9^) in the discovery meta-analysis and at two loci in the replication cohort. The two loci identified in the latter cohort were associated with the analogous glycan traits in the former cohort as detailed in the subsection “Replication of our findings”. Both UPLC and MS methods for quantitation of *N*-glycans were found to be amenable to GWA studies. Since our UPLC study gave a considerably greater yield of significant findings in comparison to MS study, the majority of our results section focuses on the findings from the discovery population cohort, which was studied using the UPLC method.

Among the nine loci that passed the genome-wide significance threshold, four contained genes encoding glycosyltransferases (*ST6GAL1*, *B4GALT1*, *FUT8* and *MGAT3*), while the remaining five loci contained genes that have not been implicated in protein glycosylation previously (*IKZF1, IL6ST-ANKRD55, ABCF2-SMARCD3, SUV420H1-CHKA* and *SMARCB1-DERL3*). As a rule, the implicated genes were associated with several *N*-glycan traits. The explanation and notation of the 77 N-glycan measures is presented in Table S1. It comprises 23 directly measured quantitative IgG glycosylation traits (shown in Figure 1) and 54 derived traits. Descriptive statistics of these measures in the discovery cohorts are presented in Table S2. GWA analysis was performed in each of the populations separately and the results were combined in an inverse-variance weighted meta-analysis. Summary data for each gene region showing genome-wide association (p<27.2×10^−9^) or found to be strongly suggestive (2.27×10^−9^<p<5×10^−8^) are presented in Table 1. Summary data for all single-nucleotide polymorphisms (SNPs) and traits with suggestive associations (p<1×10^−5^) are presented in Table S3, with population-specific and pooled genomic control (GC) factors reported in Table S4.

**Table 1:** A complete list of genetic markers that showed genome-wide significant (P<2.27E-9) or strongly suggestive (P≤5E-08) association with glycosylation of Immunoglobulin G analysed by UPLC in the discovery meta-analysis.

Chr.	SNP with lowest P-value	Lowest P-value	Effect size* (s.e.)	MAF	Interval size, kb	nHits	nTraits	Genes in the interval	Trait with lowest P-value+	Other Associated Traits+
**Genome-wide Significant**										
3	rs11710456	6.12E-75	0.64 (0.04)	0.30	14.2	20	14	*ST6GAL1*	IGP29	IGP14$, IGP15, IGP17, IGP23, IGP24, IGP26, IGP28, IGP30, IGP31$, IGP32, IGP35$, IGP37$, IGP38$
5	rs17348299	6.88E-11	0.29 (0.04)	0.16	16.1	4	6	*IL6ST-ANKRD55*	IGP53	IGP3, IGP13, IGP43, IGP55, IGP57
7	rs6421315	1.87E-13	0.23 (0.03)	0.37	21.4	11	13	*IKZF1*	IGP63	IGP2$, IGP6$, IGP42$, IGP46$, IGP58, IGP59, IGP60, IGP62, IGP67$, IGP70$, IGP71$, IGP72
7	rs1122979	2.10E-10	0.31 (0.05)	0.12	62.3	3	4	*ABCF2-SMARCD3*	IGP2	IGP5, IGP42, IGP45
9	rs12342831	2.70E-11	−0.24 (0.04)	0.26	60.1	28	11	*B4GALT1*	IGP17	IGP13, IGP24, IGP26, IGP36$, IGP37$, IGP38$, IGP39$, IGP40$, IGP53, IGP57
11	rs4930561	8.88E-10	0.19 (0.03)	0.49	58.7	5	2	*SUV420H1*	IGP41	IGP1
14	rs11847263	1.08E-22	−0.31 (0.03)	0.39	17.1	167	12	*FUT8*	IGP59	IGP2$, IGP6$, IGP11$, IGP42$, IGP46$, IGP51$, IGP58, IGP60, IGP61, IGP63, IGP65
22	rs2186369	8.63E-17	0.35 (0.04)	0.19	49.4	10	20	*SMARCB1-DERL3*	IGP72	IGP9$, IGP10$, IGP14$, IGP39$, IGP40$, IGP49$, IGP50$, IGP62, IGP63, IGP64, IGP66$, IGP67$, IGP68$, IGP69$, IGP70$, IGP71$, IGP74$, IGP75$, IGP76
22	rs909674	9.66E-25	0.34 (0.03)	0.30	27.9	60	17	*SYNGR1-TAB1-MGAT3-CACNA1I*	IGP40	IGP5, IGP9, IGP22$, IGP34, IGP39, IGP45, IGP49, IGP62$, IGP63$, IGP64$, IGP66, IGP67, IGP68, IGP70, IGP71, IGP72$
**Strongly Suggestive**										
6	rs9296009	3.79E-08	−0.21 (0.04)	0.20	–	1	1	*PRRT1*	IGP23	–
6	rs1049110	1.64E-08	0.19 (0.03)	0.35	32.3	1	2	*HLA-DQA2, HLA-DQB2*	IGP42	IGP2
6	rs404256	7.49E-09	−0.21 (0.04)	0.44	–	1	1	*BACH2*	IGP7	–
7	rs2072209	1.16E-08	−0.37 (0.07)	0.06	–	1	1	*LAMB1*	IGP69	–
9	rs4878639	3.51E-08	−0.20 (0.04)	0.26	14.4	1	1	*RECK*	IGP17	–
12	rs12828421	4.48E-08	−0.18 (0.03)	0.49	29.6	2	1	*PEX5*	IGP41	–
17	rs7224668	3.33E-08	0.17 (0.03)	0.48	45.9	2	1	*SLC38A10*	IGP31	–

Interval: size (kb) of the genomic interval containing SNPs with R^2^> = 0.6 with top associated SNP; nHits: number of SNPs with GW-significant association; nTraits: number of IgG glycosylation traits associated with the region at GW-significant level;

* effect size is in z-score units after adjustment for sex, age and first 3 principal components.

+ Description of the traits provided in Table S1;

$ the SNP effect in opposite direction to most significant trait.

**Figure 2:**
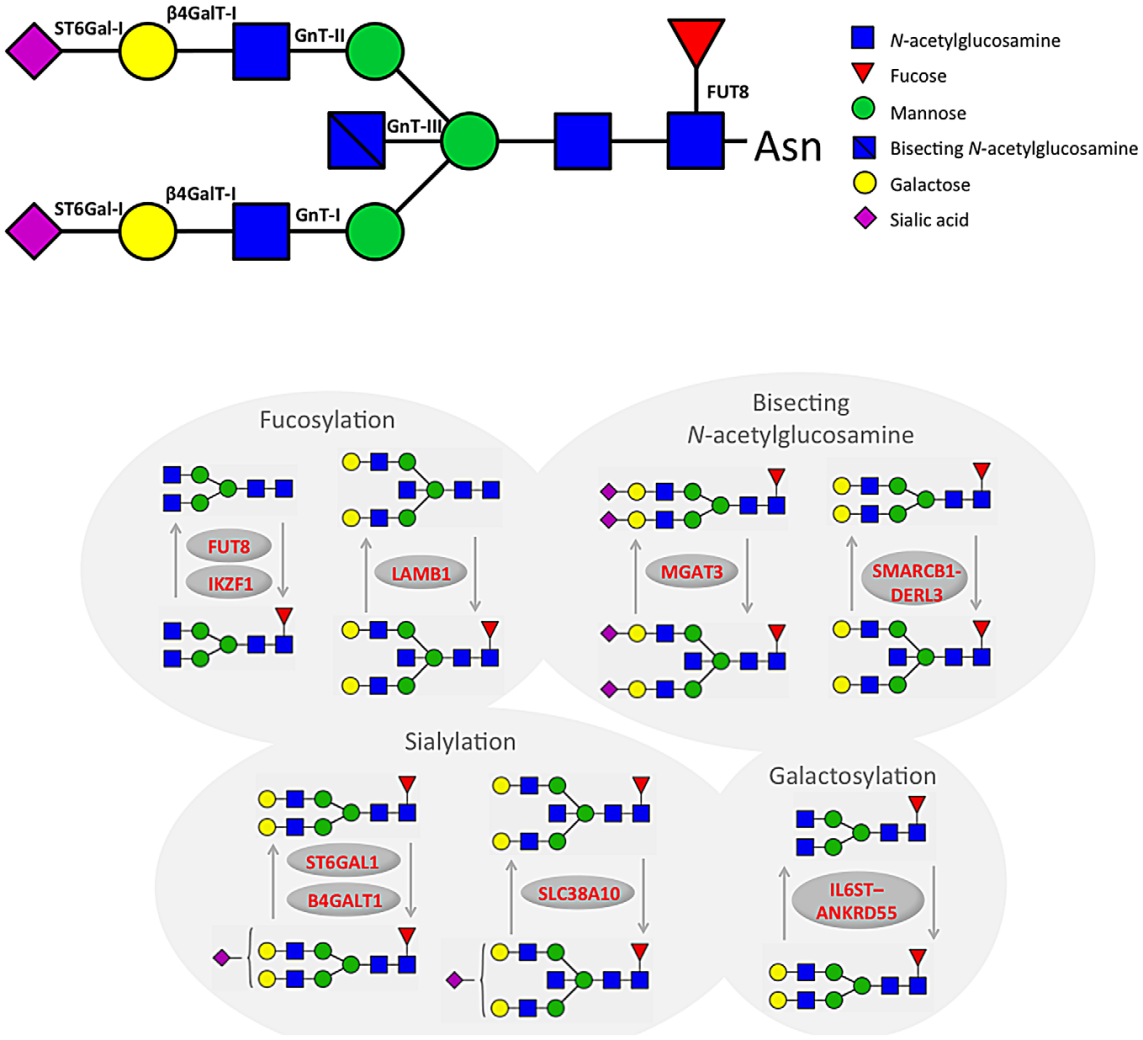
A summary of changes to IgG N-glycan structures that were associated with 16 loci identified through GWA study.

The most statistically significant association was observed in a region on chromosome 3 containing the gene *ST6GAL1* (Table 1, Figure S1A). *ST6GAL1* codes for the enzyme sialyltransferase 6 which adds sialic acid to various glycoproteins including IgG glycans (Figure 2), and is therefore a highly biologically plausible candidate. In this region of about 70 kilobases (kb) we identified 37genome-wide significant SNPs associated with 14 different IgG glycosylation traits, generally reflecting sialylation of different glycan structures (Table 1). The strongest association was observed for the percentage of monosialylation of fucosylated digalactosylated structures in total IgG glycans (IGP29, see Figure 1 and Table S1 for notation), for which a SNP rs11710456 explained 17%, 16%, 18% and 3% of the trait variation for CROATIA-Vis, CROATIA-Korcula, ORCADES and NSPHS respectively (meta-analysis p = 6.12×10^−75^). NSPHS had a very small sample size in this analysis (N = 179) and may not provide an accurate portrayal of the variance explained in this particular population (estimated as 3%). Although the allele frequency is similar between all populations, in the forest plot (Figure S1A) although NSPHS does overlap with the other populations, the 95% CI is much larger. It is also possible that there are population-specific genetic and/or environmental differences in NSPHS that are affecting the amount of variance explained by this SNP. After analysis conditioning on the top SNP (rs11710456) in this region, the SNP rs7652995 still reached genome-wide significance (p = 4.15×10^−13^). After adjusting for this additional SNP, the association peak was completely removed. This suggests that there are several genetic factors underlying this association. Conditional analysis of all other significant and suggestive regions resulted in the complete removal of the association peak.

We also identified 28 SNPs showing genome-wide significant associations with 11 IgG glycosylation traits (2.70×10^−11^<p<4.73×10^−8^) at a locus on chromosome 9 spanning over 60 kb (Figure S1B). This region includes *B4GALT1*, which codes for the galactosyltransferase responsible for the addition of galactose to IgG glycans (Figure 2). The glycan traits showing genome-wide association included the percentage of FA2G2S1 in the total fraction (IGP17), the percentage of FA2G2 in the total and neutral fraction (IGP13, IGP53), the percentage of sialylation of fucosylated structures without bisecting GlcNAc (IGP24, IGP26), the percentage of digalactosylated structures in the total neutral fraction (IGP57) and, in the opposite direction, the percentage of bisecting GlcNAc in fucosylated sialylated structures (IGP36–IGP40).

A large (541 kb) region on chromosome 14 harbouring the *FUT8* gene contained 167 SNPs showing significant associations with 12 IgG glycosylation traits reflecting fucosylation of IgG glycans (Figure S1C). *FUT8* codes for fucosyltransferase 8, an enzyme responsible for the addition of fucose to IgG glycans (Figure 2). The strongest association (1.08×10^−22^<p<2.60×10^−17^) was observed with the percentage of A2 glycans in total and neutral fractions (IGP2, IGP42) and for derived traits related to the proportion of fucosylation (IGP58, IGP59 and IGP61; all in the opposite direction). In summary, SNPs at the *FUT8* locus influence the proportion of fucosylated glycans, and, in the opposite direction, the percentages of A2, A2G1 and A2G2 glycans which are not fucosylated.

On chromosome 22, two loci were associated with IgG glycosylation. The first region, containing *SYNGR1-TAB1-MGAT3-CACNA1I* genes, spans over 233 kb. This region harboured 60 SNPs showing genome-wide significant association with 17 IgG glycosylation traits (Figure S1D). Association was strongest between SNP rs909674 and the incidence of bisecting GlcNAc in all fucosylated disialylated structures (IGP40, p = 9.66×10^−25^) and the related ratio IGP39 (p = 8.87×10^−24^). In summary, this locus contained variants influencing levels of fucosylated species and the ratio between fucosylated (especially disialylated) structures with and without bisecting GlcNAc (Figure 2). Since *MGAT3* codes for the enzyme *N*-acetylglucosaminyltransferase III (beta-1,4-mannosyl-glycoprotein-4-beta-*N*-acetylglucosaminyltransferase), which is responsible for the addition of bisecting GlcNAc to IgG glycans, this gene is the most biologically plausible candidate.

Bioinformatic analysis of known and predicted protein-protein interactions using String 9.0 software (http://string-db.org/) showed that interactions between the clusters of *FUT8*-*B4GALT1*-*MGAT3* genes and *ST6GAL1*-*B4GALT1*-*MGAT3* genes had high confidence score: *FUT8-B4GALT1* of 0.90; *FUT8-MGAT3* of 0.95; *ST6GAL1-B4GALT1* of 0.90; and *ST6GAL1-MGAT3* of 0.73. The glycosyltranferase genes at the four GWAS loci - *ST6GAL1*, *B4GALT1*, *FUT8*, and *MGAT3* – are responsible for adding sialic acid, galactose, fucose and bisecting GlcNAc to IgG glycans, thus demonstrating the proof of principle that a single protein glycosylation GWAS approach can identify biologically important glycan pathways and their networks. Interestingly, *ST6GAL1* has been previously associated with Type 2 diabetes [20], *MGAT3* with Crohn's disease [21], primary biliary cirrhosis [22] and cardiac arrest [23], and FUT8 with multiple sclerosis, blood glutamate levels [24] and conduct disorder [25] (Table 2). We have recently shown changes in plasma N-glycan profile between patients with attention-deficit hyperactivity disorder (ADHD), autism spectrum disorders and healthy controls, and identified loci influencing plasma *N*-glycome with pleiotropic effects on ADHD [26], [27].

**Table 2:** An analysis of pleiotropy between loci associated with IgG glycans and previously reported disease/trait susceptibility loci, with linkage disequilibrium computed between the most significantly associated SNPs.

Gene	IgG Glycan Top SNP	Disease	Top Disease SNP	Risk Allele	P-Value	Reference	Ancestry	HapMap 2		1000G Pilot 1	
								R^2^	D′	R^2^	D′
IKZF1	rs6421315	SLE	rs921916	C	2.00E-06	Gateva et al Nat Genet 2009	European	0.021	0.388	0.070	0.771
		SLE	rs2366293	G	2.33E-09	Cunninghame Graham et al PLoS Genet 2011	European	0.030	0.484	0.057	0.748
		SLE	rs4917014	A	3.00E-23	Han et al Nat Genet 2009	Han Chinese	0.001	0.040	0.053	0.277
		ALL	rs11978267	G	8.00E-11	Trevino et al Nat Genet 2009	European	0.002	0.047	0.012	0.130
		ALL	rs4132601	C	1.00E-19	Papeammanuil et al Nat Genet 2009	European	0.002	0.047	0.012	0.130
		Hippocampal atrophy (AD qt)	rs10276619	–	3.00E-06	Potkin et al PLoS One 2009	European	0	0.005	0	0.013
		Total ventricular volume (AD qt)	rs7805803	–	9.00E-06	Furney et al Mol Psychiatry 2010	European	0.071	0.280	0.087	0.332
		Crohn's disease	rs1456893	A	5.00E-09	Barrett et al Nat Genet 2008	European	0.011	0.117	0	0.007
		Mean corpuscular volume	rs12718597	A	5.00E-13	Ganesh et al Nat Genet 2009	European	0.018	0.181	0.019	0.161
		Malaria	rs1451375	–	6.00E-06	Jallow et al Nat Genet 2009	Gambian	0.014	0.204	0.002	0.097
		Systemic sclerosis	rs1240874	–	1.00E-06	Gorlova et al PLoSGenet 2011	European	–	–	–	–
		T1D	rs10272724	C	1.10E-11	Swafford et al Diabetes 2011	European	0.002	0.047	0.012	0.130
ST6GAL1	rs11710456	Drug-induced liver injury (flucloxacillin)	rs10937275	–	1.00E-08	Daly et al Nat Genet 2009	European	0	0.048	0.017	1
		T2D	rs16861329	G	3.00E-08	Kooner et al Nat Genet 2011	South Asian	0.011	0.221	0.000	0.005
IL6ST-ANKRD55	rs17348299	Rheumatoid arthritis	rs6859219	C	1.00E-11	Stahl et al Nat Genet 2010	European	0.012	0.487	0.044	1.000
LAMB1	rs2072209	Ulcerative colitis	rs2158836	A	7.00E-06	Silverberg et al Nat Genet 2009	European	0.027	0.716	0.055	1.000
		Ulcerative colitis	rs4598195	A	8.00E-08	McGovern et al Nat Genet 2010	European	0.071	0.807	0.115	1.000
		Ulcerative colitis	rs886774	G	3.00E-08	Barrett et al Nat Genet 2009	European	0.031	0.733	0.067	1.000
		Ulcerative colitis	rs4510766	A	2.00E-16	Anderson et al Nat Genet 2011	European	–	–	0.107	1.000
		Ulcerative colitis	rs4730276	–	9.00E-06	Silverberg et al Nat Genet 2009	European	–	–	0.038	0.534
		Ulcerative colitis	rs4730273	–	5.00E-06	Silverberg et al Nat Genet 2009	European	0.032	1.000	0.027	0.931
		Ulcerative colitis	rs2108225	A	1.00E-07	Asano et al Nat Genet 2009	Japanese	0.024	0.548	0.017	0.482
FUT8	rs11847263	N-Glycans (DG6)	rs10483776	G	1.00E-08	Lauc et al PLoS Genet 2010	European	0.369	0.864	0.416	0.758
		N-Glycans (DG1)	rs7159888	A	3.00E-18	Lauc et al PLoS Genet 2010	European	0.714	1	0.727	1
		Conduct disorder (symptom count)	rs1256531	–	4.00E-06	Dick et al Mol Psychiatry 2010	European, African, other	0.092	0.796	0.071	1
		Waist Circumference	rs7158173	–	4.00E-06	Polasek et al Croat Med J 2009	European	0.011	0.182	0.002	0.081
		Multiple Sclerosis - brain glutamate levels	rs8007846	–	9.00E-06	Baranzini et al Brain 2010	American	0.196	0.571	0.261	0.692
SYNGR1-TAB1-MGAT3-CACNA1I	rs909674	Sudden cardiac arrest	rs54211	–	8.00E-07	Aouizerat et al BMC Car Diso 2011	European	0.053	0.362	0.043	0.360
		Primary biliary cirrhosis	rs968451	T	1.00E-09	Mells et al Nat Genet 2011	European	0.041	0.682	0.080	1
		Crohn's disease	rs2413583	C	1.00E-26	Franke et al Nat Genet 2010	European	0.043	0.313	0.053	0.292
SMARCB1-DERL3	rs2186369	GGT	rs2739330	T	2.00E-09	Chambers et al Nat Genet 2011	European	0.009	0.255	0	0.012
PRRT1	rs9296009	Nodular sclerosis Hodgkin lymphoma	rs204999	–	8.00E-18	Cozen et al Blood 2012	European	0.125	1	–	–
		Phospholipid levels	rs1061808	–	8.00E-10	Demirkan et al PLoS Genet 2012	European	0.137	0.626	–	–
HLA-DQA2 - HLA-DQB2	rs1049110	SLE	rs2301271	T	2.00E-12	Chung et al PLoS Genet 2011	European	0.967	1	–	–
		Hepatitis B	rs7453920	G	6.00E-28	Mbarek et al Hum Mol Genet 2011	Japanese	0.967	1	–	–
		Narcolepsy	rs2858884	A	3.00E-08	Hor et al Nat Genet 2010	European	0.193	1	–	–
BACH2	rs404256	Graves' disease	rs370409	T	2.00E-06	Chu et al Nat Genet 2011	Chinese	0.010	0.187	0.009	0.166
		Celiac disease	rs10806425	A	4.00E-10	Dubois et al Nat Genet 2010	European	0.005	0.103	0.006	0.09
		T1D	rs3757247	A	1.00E-06	Grant et al Diabetes 2009	European	0.039	0.196	0.042	0.204
		T1D	rs11755527	G	3.00E-08	Plagnol et al PLoSGenet 2011	European	0.031	0.186	0.031	0.179
		T1D	rs11755527	–	5.00E-08	Barrett et al Nat Genet 2009	European	0.031	0.186	0.031	0.179
		T1D	rs11755527	G	5.00E-12	Cooper et al Nat Genet 2008	European	0.031	0.186	0.031	0.179
		Crohn's disease	rs1847472	G	5.00E-09	Franke et al Nat Genet 2010	European	0	0.011	0.009	0.124
		Multiple Sclerosis	rs12212193	G	4.00E-08	Sawcer et al Nature 2011	European	0.001	0.036	0.027	0.166
SLC38A10	rs7224668	Longevity	rs10445407	–	1.00E-06	Yashin et al Aging 2010	European	0.714	1	0.692	1

Associations are those found in the GWAS Catalog track of USCS Genome browser (accessed 04/07/2012) and LD has been calculated using SNAP (http://www.broadinstitute.org/mpg/snap/Johnson, A. D., Handsaker, R. E., Pulit, S., Nizzari, M. M., O'Donnell, C. J., de Bakker, P. I. W. SNAP: A web-based tool for identification and annotation of proxy SNPs using HapMap *Bioinformatics, 2008 24(24):2938–2939*).

### Novel candidate genes involved with N-glycosylation

In addition to four loci containing genes for enzymes known to be involved in IgG glycosylation, our study also found five unexpected associations showing genome-wide significance. In the second region on chromosome 22 we observed genome-wide significant associations of 10 SNPs with 20 IgG glycosylation traits. The region spans 49 kb and contains the genes *SMARCB1-DERL3* (Figure S1E). The strongest associations (8.63×10^−17^<p<3.00×10^−13^) were observed between SNP rs2186369 and the percentage of FA2[6]BG1 in total and neutral fractions (IGP9, IGP49) and levels of fucosylated structures with bisecting GlcNAc (IGP66, IGP68, IGP70, IGP71 in the same direction and IGP72 in the opposite direction). Thus, the *SMARCB1-DERL3* locus appears to specifically influence levels of fucosylated monogalactosylated structures with bisecting GlcNAc (Figure 2). *DERL3* is a promising functional candidate, because it encodes a functional component of endoplasmic reticulum (ER)-associated degradation for misfolded luminal glycoproteins [28]. However, *SMARCB1* is also known to be important in antiviral activity, inhibition of tumour formation, neurodevelopment, cell proliferation and differentiation [29]. The region has also been implicated in the regulation of γ-glutamyl-transferase (GGT) [30] (Table 2).

A locus on chromosome 7 spanning 26kb contained 11 SNPs showing genome-wide significant associations with 13 IgG glycosylation traits (Figure S1F). The strongest association (p = 1.87×10^−13^) was observed between SNP *rs6421315* located in *IKZF1* and the percentage of fucosylation of agalactosylated structures without bisecting GlcNAc (IGP63). Thus, SNPs at this locus influence the percentage of non-fucosylated agalactosylated glycans, the fucosylation ratio in agalactosylated glycans (in opposite directions for glycan species with and without bisecting GlcNAc), and the ratio of fucosylated structures with and without bisecting GlcNAc (Figure 2). The *IKZF1* gene encodes the DNA-binding protein Ikaros, acting as a transcriptional regulator and associated with chromatin remodelling. It is considered to be the important regulator of lymphocyte differentiation and has been shown to influence effector pathways through control of class switch recombination [31], thus representing a promising functional candidate [32]. There is overwhelming evidence that *IKZF1* variants are associated with childhood acute lymphoblastic leukaemia [33], [34] and several diseases with an autoimmune component: systemic lupus erythematosus (SLE) [35]–[37], type 1 diabetes [38], [39], Crohn's disease [40], systemic sclerosis [41], malaria [42] and erythrocyte mean corpuscular volume [43] (Table 2).

SNPs at several other loci also showed genome-wide significant association with a number of different IgG glycosylation traits (Figure S1G–S1P). Chromosome 5 SNP rs17348299, located in *IL6ST-ANKRD55* was significantly associated (6.88×10^−11^<p<2.39×10^−9^) with six IgG glycosylation traits, including FA2 and FA2G2 in total and neutral fractions (IGP3, IGP13, IGP43, IGP53) and the percentage of agalactosylated and digalactosylated structures in total neutral IgG glycans (IGP55, IGP57) (Figure 2). The protein encoded by *IL6ST* is a signal transducer shared by many cytokines, including interleukin 6 (IL6), ciliary neurotrophic factor (CNTF), leukaemia inhibitory factor (LIF), and oncostatin M (OSM). Variants in *IL6ST* have been associated with rheumatoid arthritis and multiple myeloma, but also with components of metabolic syndrome [44]–[46].

The chromosome 7 SNP rs2072209 located in *LAMB1* was strongly suggestively associated with the percentage of fucosylation of digalactosylated (with bisecting GlcNAc) structures (IGP69; p = 1.16×10^−8^) (Figure 2). *LAMB1* (laminin beta 1) is a member of a family of extracellular matrix glycoproteins that are the major non-collagenous constituent of basement membranes. It is thought to mediate the attachment, migration and organization of cells into tissues during embryonic development by interacting with other extracellular matrix components. It has been associated with ulcerative colitis in several large-scale studies in European and Japanese populations, suggesting that changes in the integrity of the intestinal epithelial barrier may contribute to the pathogenesis of the disease [47]–[51] (Table 2).

Another particularly interesting finding was the suggestive association between rs404256 in the *BACH2* gene on chromosome 6 and IGP7, defined through proportional contribution of FA2[6]G1 in all IgG glycans (p = 7.49×10^−9^). *BACH2* is B-cell-specific transcription factor that can act as a suppressor or promoter; among many known functions, it has been shown to “orchestrate” transcriptional activation of B-cells, modify the cytotoxic effects of anticancer drugs and regulate IL-2 expression in umbilical cord blood CD4^+^ T cells [52]. *BACH2* has been previously associated with a spectrum of diseases with autoimmune component: type 1 diabetes [53]–[56], Graves' disease [57], celiac disease [58], Crohn's disease [21] and multiple sclerosis [59] (Table 2).

The chromosome 11 SNP rs4930561 located in the *SUV420H1-CHKA* gene was associated with percentage of FA1 in neutral (IGP41; p = 8.88×10^−10^) and total (IGP1; p = 1.30×10^−8^) fractions of IgG glycans. *SUV420H1* codes for histone-lysine N-methyltransferase which specifically trimethylates lysine 20 of histone H4 and could therefore affect activity of many different genes; it is thought to be involved in proviral silencing in somatic and germ line cells through epigenetic mechanisms [60]. *CHKA* has a key role in phospholipid biosynthesis and may contribute to tumour cell growth. We recently reported a number of strong associations between lipidomics and glycomics traits in human plasma [61]. Thus, an enzyme involved in phospholipid synthesis is also a possible candidate because the lipid environment is known to affect glycosyltransferases activity [61].

Three further loci were identified as strongly suggestive through GWAS and deserve attention for their possible pleiotropic effects. SNP rs9296009 in *PRRT1* (proline-rich transmembrane protein 1) was associated with IGP23 (p = 3.79×10^−08^) while variants in *PRRT1* previously showed associations with nodular sclerosis and Hodgkin lymphoma [62]. Moreover, rs1049110 in *HLA-DQA2-HLA-DQB2* was associated with IGP2 and IGP42 (p = 1.64×10^−08^ and 4.44×10^−08^, respectively). This SNP is in nearly complete linkage disequilibrium with two other SNPs in this region that have previously been associated with SLE and hepatitis B [63] (Table 2). Another SNP in this region has been linked with narcolepsy [64]. Finally, rs7224668 in SLC38A10, a putative sodium-dependent amino acid/proton antiporter, showed significant association with IGP31 (p = 3.33×10^−08^). Although the function of this gene is not understood, it has been associated with autism and longevity [65], [66].

The remaining three signals implicated *ABCF2-SMARCD3* region (rs1122979 was associated with IGP 2, 5, 42, 45, with p-value ranging between 2.10×10^−10^<p<1.89×10^−9^), *RECK* (rs4878639 was suggestively associated with IGP17; p = 3.51×10^−8^) and *PEX5* (rs12828421 suggestively associated with IGP41; p = 4.48×10^−8^). The function of *ABCF2* (ATP-binding cassette, sub-family F, member 2) is not well understood. *SMARCD3* stimulates nuclear receptor mediated transcription; it belongs to the neural progenitors-specific chromatin remodelling complex (npBAF complex) and the neuron-specific chromatin-remodelling complex (nBAF complex). *RECK* is known to be a strong suppressor of tumour invasion and metastasis, regulating metalloproteinases which are involved in cancer progression. *PEX 5* binds to the C-terminal PTS1-type tripeptide peroxisomal targeting signal and plays an essential role in peroxisomal protein import (www.genecards.org).

### Results from an independent cohort using MS quantitation method

The parallel effort in the outbred Leiden Longevity Study (LLS) was based on a different *N*-glycan quantitation method (MS). While UPLC groups glycans according to structural similarities, MS groups them by mass. Furthermore, MS analysis focused on Fc glycans while UPLC measures both Fc and Fab glycans, thus traits measured by the two methods could not have been directly compared. Glycosylation patterns of IgG1 and IgG2 were investigated by analysis of tryptic glycopeptides, with six glycoforms per IgG subclass measured. The intensities of all glycoforms were related to the monogalactosylated, core-fucosylated biantennary species, providing five relative intensities registered per IgG subclass (Tables S5 and S6). The analysis identified two loci as genome-wide significant - implicating *MGAT3* (p = 1.6×10^−10^ for G1FN, analogous to UPLC IGP9; p = 3.12×10^−8^ for G0FN, analogous to UPLC IGP5), and *B4GALT1* (p = 5.4×10^−8^ for G2F, analogous to UPLC IGP13) confirming GWAS signals in the discovery meta-analysis.

### Replication of our findings

We then sought a separate independent replication of the other 14 genome-wide significant and strongly suggestive signals identified in the discovery analysis, which was performed in the LLS cohort, appreciating that the quantitated *N*-glycan traits do not exactly match between the two cohorts. SNPs were chosen for replication based on initial meta-analysis results of genotype data prior to imputed analysis. All five traits measured in LLS cohort were tested for association with all the selected SNPs (Table S6). We were able to reproduce association to *ST6GAL1* (p = 8.1×10^−7^ for G2F, substrate for sialyltransferase) and *SMARCB1-DERL3* (p = 1.6×10^−7^ for G1N, analogous to UPLC IGP9). Weaker, though nominally significant associations were confirmed at *IKZF1* (p = 2.3×10^−3^ for G1N), *SLC38A10* (p = 4.8×10^−3^ for G2N), *IL6ST-ANKRD55* (p = 1.3×10^−2^ for G0N) and *ABCF2-SMARCD3* (p = 2.7×10^−2^ for G2N). The fact that we did not replicate associations at the other 8 loci was not unexpected, because those 8 loci showed association with UPLC-measured *N*-glycan traits that do not compare to any of the traits measured by MS (see Table S5 for comparison of MS and UPLC traits).

### Functional experiment: Ikzf1 haplodeficiency results in altered N-glycosylation of IgG

*IKZF1* is considered to be the important regulator governing differentiation of T cells into CD4+ and CD8+ T cells [67]. Since glycan traits associated with *IKZF1* were related to the presence and absence of core-fucose and bisecting GlcNAc, we analysed the promoter region of *MGAT3* (codes for enzyme that adds bisecting GlcNAc to IgG glycans) *in silico* and identified two binding sites for *IKZF1* that were conserved between humans and mice, while recognition sites for *IKZF1* were not found in the promoter region of *FUT8* (which codes for an enzyme that adds core-fucose to IgG glycans). Since the promoter regions of *MGAT3* were conserved between humans and mice, we used *Ikzf1* knockout mice [68] as a model to study the effects of *IKZF1* deficiency on IgG glycosylation. IgG was isolated from the plasma of 5 heterozygous knockout mice and 5 wild-type controls. The summary of the results of IgG glycosylation analysis is presented in Table 3, while complete results are presented in Table S7. We observed a number of alterations in glycome composition that were all consistent with the role of *IKZF1* in the down-regulation of fucosylation and up-regulation of the addition of bisecting GlcNAc to IgG glycans; 12 out of 77 IgG N-glycans measures showed statistically significant difference (p<0.05) between wild type and heterozygous *Ikzf1* knock-outs, where 5 mice from each group were compared (Table 3). The empirical version of Hotelling's test demonstrated global significance (p = 0.03) of difference between distributions of IgG glycome between wild type and *Ikzf1* knock-out mice, where 5 mice from each group were compared. While the tests for differences between individual glycome measurements did not reach strict statistical significance after conservative Bonferroni correction (p = 0.05/77 = 0.0006), we observed that 12 out of 77 (15%) IgG N-glycans measures showed nominally significant difference (p<0.05) between wild type and heterozygous *Ikzf1* knock-outs (Table 3). Significant results from the global difference test ensure that difference between the two groups does exist, and it is most likely due to the difference between (at least some of) the measurements which demonstrated nominal significance. Observed alterations in glycome composition were all consistent with the role of *IKZF1* in the down-regulation of fucosylation and up-regulation of the addition of bisecting GlcNAc to IgG glycans.

**Table 3:** Twelve groups of IgG N-glycans (of 77 measured) that showed nominally significant difference (p<0.05) in observed values between 5 mice that were heterozygous *Ikzf1* knock-outs (Neo) and 5 wild-type controls (wt).

Increased N-glycans					
N-glycan group code	N-glycan trait	Mean (Neo)	Mean (wt)	Mean(Neo)/Mean(wt)	p-value*
IGP8	GP9 - FA2[3]G1	8.91	7.44	1.20	3.54E-03
IGP48	GP9n – GP9/GPn*100	11.71	10.34	1.13	1.41E-02
IGP64	% FG1n/G1n	98.47	97.53	1.01	2.63E-02

Decreased N-glycans					
N-glycan group code	N-glycan trait	Mean (Neo)	Mean (wt)	Mean(Neo)/Mean(wt)	p-value*
IGP9	GP10 - FA2[6]BG1	0.13	0.17	0.76	2.32E-03
IGP10	GP11 - FA2[3]BG1	0.34	0.62	0.55	4.74E-02
IGP19	GP20 – (undetermined)	12.29	15.07	0.82	7.62E-03
IGP23	GP24 - FA2BG2S2	1.27	1.50	0.85	3.20E-02
IGP37	FBS1/FS1	0.12	0.18	0.67	4.93E-02
IGP38	FBS1/(FS1+FBS1)	0.10	0.15	0.67	4.84E-02
IGP49	GP10n – GP10/GPn*100	0.17	0.24	0.71	1.71E-03
IGP50	GP11n – GP11/GPn*100	0.44	0.87	0.51	4.27E-02
IGP68	% FBG1n/G1n	1.15	2.05	0.56	2.76E-02

The global difference test was significant (p = 0.03). ^*^t-test for equality of means (2-tailed).

### Investigating the biomarker potential of IgG N-glycans in Systemic Lupus Erythematosus (SLE)

Given that *IKZF1* has been convincingly associated with SLE in previous studies [35]–[37], and that functional studies in heterozygous knock-out mice in our study showed clear differences in profiles of several IgG *N*-glycan traits, we explored an intriguing hypothesis: whether the same IgG *N*-glycan traits that were significantly affected in *Ikzf1* knock-out mice could be demonstrated to differ between human SLE cases and controls. If this were true, then pleiotropy between the effects of *IKZF1* on SLE and on IgG *N*-glycans in human plasma, revealed by independent GWA studies, would lead to a discovery of a novel class of biomarkers of SLE – IgG *N*-glycans – which could possibly extend their usefulness in prediction of other autoimmune disorders, cancer and neuropsychiatric disorders, through the same mechanism.

To test this hypothesis, we measured IgG *N*-glycans in 101 SLE cases and 183 matched controls (typically two controls per case), recruited in Trinidad (see materials and methods for further details). Table 4 shows the results of the measurements: for 10 of 12 *N*-glycan traits chosen on a basis of the experiments in mice (Table 3). The entire dataset for all glycans can be found in Table S8. There was a statistically significant difference (p<0.05) between SLE cases and controls, which was generally not the case with other groups of *N*-glycans (data not shown). Moreover, the significance of the difference was striking in some cases, e.g. p<10^−14^ for IGP48, p<10^−13^ for IGP8, and p<10^−6^ for IGP64. Furthermore, the differences in the direction of effect in mice were strikingly preserved in humans (Table 4). The most significant differences observed across all 77 IgG *N*-glycans measurements between SLE cases and controls (Table 4) were overlapping well with the 12 *N*-glycan groups that were significantly changed in functional experiments in *Ikzf1* knock-out mice.

**Table 4:** Groups of IgG N-glycans from Table 3 that showed statistically significant difference in observed values (corrected by sex, age, and African admixture) between 101 Afro-Caribbean cases with SLE and 183 controls.

Decreased N-glycans					
N-glycan group code	N-glycan trait	Mean (SLE)	Mean (controls)	Mean(SLE)/Mean(controls)	p-value*
IGP8	GP9 - FA2[3]G1	6.67	8.03	0.83	1.86E-14
IGP48	GP9n – GP9/GPn*100	9.09	11.06	0.82	6.72E-15
IGP64	% FG1n/G1n	80.93	83.22	0.97	5.07E-07
IGP19	GP20 – (undetermined)	0.73	0.80	0.91	4.87E-02

Increased N-glycans					
N-glycan group code	N-glycan trait	Mean (SLE)	Mean (controls)	Mean(SLE)/Mean(controls)	p-value*
IGP9	GP10 - FA2[6]BG1	4.58	4.13	1.10	4.37E-04
IGP23	GP24 - FA2BG2S2	3.00	2.65	1.13	1.67E-03
IGP37	FBS1/FS1	0.20	0.18	1.11	5.08E-03
IGP38	FBS1/(FS1+FBS1)	0.17	0.15	1.13	4.83E-03
IGP49	GP10n – GP10/GPn*100	6.24	5.70	1.09	1.39E-03
IGP68	% FBG1n/G1n	17.34	15.30	1.13	2.19E-06

* t-test for equality of means (2-tailed).

To strengthen our findings and control for possible bias, we repeated the analysis excluding all the cases on corticosteroid treatment at the time of interview (77/101) and subsequently all the cases that were not on corticosteroid treatment at the time of interview (24/101). Although the power of the analysis decreased due to reduced number of cases, the results did not change and they remained highly statistically significant. We also hypothesized that the observed glycan changes may not be specific to SLE, but may be caused by corticosteroid treatment, or secondary to any inflammatory process. For this reason, and in SLE cases only, we investigated whether corticosteroid treatments and/or CRP measurements, were associated with IgG *N*-glycan traits. Analysis for CRP was repeated with CRP treated as a binary variable (with cut-off value at 10 mg/L). In all these analyses, the initial results held and were not changed: the association of IgG *N*-glycans and SLE remained striking, while the association with corticosteroid treatment and CRP was not (Table S9). Finally, we also repeated the analysis adjusting for percent African admixture, as it has been reported that SLE in Afro-Caribbean population is associated with African admixture [69]. However, this adjustment only had a minor and non-systemic effect on the previous results, and the reported observations remained.

**Figure 3:**
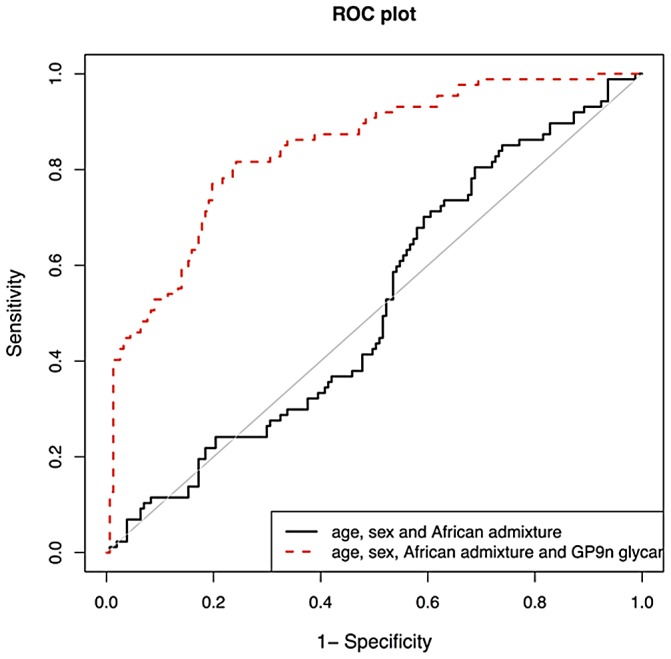
Validation of biomarker potential of IGP48 IgG N-glycan percentage in prediction of Systemic Lupus Erythematosus (SLE) in 101 Afro-Caribbean cases and 183 matched controls. As shown in the graph, age and sex do not have any predictive power for this disease, but addition of IGP48 substantially increases sensitivity and specificity of prediction, with area under receiver-operator curve increased to 0.828.

We then validated biomarker potential of IGP48, the IgG *N*-glycan trait most significantly associated with SLE status, in prediction of SLE in 101cases and 183 matched controls. We used the PredictABEL package for R (see materials and methods) [70]. As shown in Figure 3, age, sex and African admixture did not have any predictive power for this disease, but addition of IGP48 substantially increased sensitivity and specificity of prediction, with area under receiver-operator curve (AUC) increasing from 0.515 (95% confidence interval (CI): 0.441–0.590) to 0.842 (0.791–0.893). It is likely that further additions of other IgG *N*-glycans could provide even more accurate predictions. To cross-validate this result, we split our dataset with SLE cases and controls into a “training set” (2/3; 67 cases and 122 controls) and “test set” (1/3; 34 cases and 61 controls). Area under ROC-curve (AUC) was calculated for the test dataset. The whole process was repeated 1000 times, to allow computation of the mean AUC (and 95% CI) in the test datasets. Mean AUC was virtually unchanged compared to AUC obtained when using the complete dataset and no training, which suggests that the predictive power of IGP48 on SLE is very robust.

## Discussion

This study clearly demonstrates that the recent developments in high-throughput glycomics and genomics now allow identification of genetic loci that control *N*-glycosylation of a single plasma protein using a GWAS approach. This progress should allow many similar follow-up studies of genetic regulation of *N*-glycosylation of other important plasma proteins, thus bringing unprecedented insights into the role of protein glycosylation in systems biology. As a prelude to this discovery, we recently reported the results of the first GWA study of the overall human plasma *N*-glycome using the HPLC method. Although the study was of a comparable sample size (N∼2000), it only identified genome-wide associations with two glycosyltransferases and one transcription factor (*HNF1a*) [71]. We believe that the power of our initial study was reduced because *N*-glycans in human plasma originate from different glycoproteins where they have different functions and undergo protein-specific, or tissue-specific glycosylation. In this study the largest percentage of variance explained by a single association was 16–18% where as in the *N*-glycan study this was 1–6%. Furthermore, concentrations of individual glycoproteins in plasma vary in many physiological processes, introducing substantial “noise” to the quantitation of the whole-plasma *N*-glycome.

In this study we avoided both problems by isolating a single protein from plasma (IgG), which is produced by a single cell type (B lymphocytes), thus effectively excluding differential regulation of gene expression in different tissues, and the “noise” introduced by variation in plasma IgG concentration and by *N*-glycans on other plasma proteins. The only remaining “noise” in our system was the incomplete separation of some glycan structures (which co-eluted from the UPLC column) and the presence of Fab glycans on a subset of IgG molecules, but for the majority of glycan structures this “noise” was well below 10% [19]. We expected that the specificity of our phenotype and precision of the measurement provided by novel UPLC and MS methods should substantially increase the power of the study to detect genome-wide associations. Prior to analysis we could not predict which quantitation method would work better in GWA study design (UPLC vs. MS), so we used them both, each in one separate cohort of comparable sample size (N∼2000).

The UPLC method yielded many more, and much stronger, genome-wide association signals in comparison to our previous study of the total plasma *N*-glycome in virtually same sample set of examinees [27], [71]. Sixteen loci were identified in association with glycan traits with p-values<5×10^−8^ and nine reached the strict genome wide threshold of 2.27×10^−9^. The parallel study in the LLS cohort using MS quantitation has independently identified two of those 16 loci, showing genome-wide association with *N*-glycan traits. MS quantitation also allowed us to replicate 6 further loci identified in the discovery analysis, using comparable *N*-glycan traits measured by the two methods. However, in this follow-up analysis we were unable to replicate associations for the remaining 8 loci. This was not unexpected, because those glycosylation traits correspond to different fucosylated glycans; since fucosylation was not quantified by MS, the association between glycans measured by MS and those regions should not be expected.

Among the nine loci that reached genome-wide statistical significance, four involved genes encoding glycosyltransferases known to glycosylate IgG (*ST6GALI, B4GALT1, FUT8*, *MGAT3*,). The enzyme beta1,4-galactosyltransferase 1 is responsible for the addition of galactose to IgG glycans. Interestingly, variants in *B4GALT1* gene did not affect the main measures of IgG galactosylation, but rather differences in sialylation and the percentage of bisecting GlcNAc. These associations are still biologically plausible, because galactosylation is a prerequisite for sialylation, and enzymes which add galactose and bisecting GlcNAc compete for the same substrate [72]. A potential candidate for *B4GALT1* regulator is *IL6ST*, which codes for interleukin 6 signal transducer, because it showed stronger associations with the main measures of IgG galactosylation than *B4GALT1* itself. Molecular mechanisms behind this association remain elusive, but early work on *IL6* (then called *PHGF*) suggested that it may be relevant for glycosylation pathways in B lymphocytes [73].

Core-fucosylation of IgG has been intensively studied due to its role in antibody-dependent cell-mediated cytotoxicity (ADCC). This mechanism of killing is considered to be one of the major mechanisms of antibody-based therapeutics against tumours. Core-fucose is critically important in this process, because IgGs without core fucose on the Fc glycan have been found to have ADCC activity enhanced by up to 100-fold [74]. Alpha-(1,6)-fucosyltransferase (fucosyltransferase 8) catalyses the transfer of fucose from GDP-fucose to *N*-linked type complex glycopeptides, and is encoded by the *FUT8* gene. We found that SNPs located near this gene influenced overall levels of fucosylation. The directly measured IgG glycome traits most strongly associated with SNPs in the *FUT8* region consisted of A2, and, less strongly, A2G1 and A2G2. These associations are biologically plausible as these glycans serve as substrates for fucosyltransferase 8. Interestingly, SNPs located near the *IKZF1* gene influenced fucosylation of a specific subset of glycans, especially those without bisecting GlcNAc, and were also related to the ratio of fucosylated structures with and without bisecting GlcNAc. This suggests the *IKZF1* gene encoding Ikaros as a potential indirect regulator of fucosylation in B-lymphocytes by promoting the addition of bisecting GlcNAc, which then inhibits fucosylation. The analysis of IgG glycosylation in *Ikzf1* haplodeficient mice confirmed the postulated role of Ikaros in the regulation of IgG glycosylation (Table 3). The effect of *Ikzf1* haplodeficiency on IgG glycans manifested mainly in the decrease in bisecting GlcNAc on different glycan structures. The increase in fucose was observed only in a subset of structures, but since very high level of fucosylation was present in the wild type mouse (up to 99.8%), a further increase could not have been demonstrated.

Nearly all genome-wide significant loci in our study have already been clearly demonstrated to be associated with autoimmune diseases, haematologic cancers, and some of them also with chronic inflammation and/or neuropsychiatric disorders. Although the literature on those associations is extensive, we tried to highlight only those associations that were identified using genome-wide association studies in datasets independent from our study. We gave prominence to associations arising from GWA studies because they are typically replicable; GWA studies have sufficient power to detect true associations, and require stringent statistical testing and replication to avoid false positive results. They have been reviewed and summarized in Table 2. The table implies abundant pleiotropy between loci that control *N*-glycosylation (in this case, of IgG protein) and loci that have been implicated in many human diseases. Autoimmune diseases (including SLE, RA, UC and over 80 others) are generally thought to be triggered by aggressive responses of the adaptive immune system to self antigens, resulting in tissue damage and pathological sequelae [38]. Among other mechanisms, IgG autoantibodies are responsible for the chronic inflammation and destruction of healthy tissues by cross-linking Fc receptors on innate immune effector cells [75]. Class and glycosylation of IgG are important for pathogenicity of autoantibodies in autoimmune diseases (reviewed in [76]). Removal of IgG glycans leads to the loss of the proinflammatory activity, suggesting that *in vivo* modulation of antibody glycosylation might be a strategy to interfere with autoimmune processes [75]. Indeed, the removal of IgG glycans by injections of EndoS *in vivo* interfered with autoantibody-mediated proinflammatory processes in a variety of autoimmune models [75].

Results from our study suggest that IgG *N*-glycome composition is regulated through a complex interplay between loci affecting an overlapping spectrum of glycome measurements, and through interaction of genes directly involved in glycosylation and those that presumably have a “higher-level” regulatory function. SNPs at several different loci in this GWA study showed genome-wide significant associations with the same or similar IgG glycosylation traits. For example, SNPs at loci on chromosomes 9 (*B4GALT1* region) and 3 (*ST6GAL1* region) both influenced the percentage of sialylation of galactosylated fucosylated structures (without bisecting GlcNAc) in the same direction. SNPs at these loci also influenced the ratio of fucosylated monosialylated structures (with and without bisecting GlcNAc) in the opposite direction. SNPs at the locus on chromosome 9 (*B4GALT1*), and two loci on chromosome 22 (*MGAT3* and *SMARCB1-DERL3* region) simultaneously influenced the ratio of fucosylated disialylated structures with and without bisecting GlcNAc. SNPs at loci on chromosome 7 (*IKZF1* region) and 14 (*FUT8* region) influenced an overlapping range of traits: percentage of A2 and A2G1 glycans, and, in the opposite direction, the percentage of fucosylation of agalactosylated structures.

Finally, this study demonstrated that findings from “hypothesis-free” GWA studies, when targeted at a well defined biological phenotype of unknown relevance to human health and disease (such as *N*-glycans of a single plasma protein), can implicate genomic loci that were not thought to influence protein glycosylation. Moreover, unexpected pleiotropy of the implicated loci that linked them to diseases has changed this study from “hypothesis-free” to “hypothesis-driven” [77], and led us to explore biomarker potential of a very specific IgG *N*-glycan trait in prediction of a specific disease (SLE) with considerable success. To our knowledge, this is one of the first convincing demonstrations that GWA studies can lead to biomarker discovery for human disease. This study offers many additional opportunities to validate the role of further *N*-glycan biomarkers for other diseases implicated through pleiotropy.

### Conclusions

A new understanding of the genetic regulation of IgG *N*-glycan synthesis is emerging from this study. Enzymes directly responsible for the addition of galactose, fucose and bisecting GlcNAc may not have primary responsibility for the final IgG *N*-glycan structures. For all three processes, genes that are not directly involved in glycosylation showed the most significant associations: *IL6ST-ANKRD55* for galactosylation; *IKZF1* for fucosylation; and *SMARCB1-DERL3* for the addition of bisecting GlcNAc. The suggested higher-level regulation is also apparent from the differences in IgG Fab and Fc glycosylation, observed in human IgG [78], [79] and different myeloma cell lines [80], and further supported by recent observation that various external factors exhibit specific effects on glycosylation of IgG produced in cultured B lymphocytes [81].

Moreover, this study showed that it is possible to identify loci that control glycosylation of a single plasma protein using a GWAS approach, and to develop a novel class of disease biomarkers. This should lead to large advances in understanding of the role of protein glycosylation in the future. This study identified 16 genetic loci that are likely to be part of a much larger genetic network that regulates the complex process of IgG *N*-glycosylation and several further loci that show suggestive association with glycan traits and merit further study. Genetic variants in several of these genes were previously associated with a number of inflammatory, neoplastic and neuropsychiatric diseases across ethnically diverse populations, all of which could benefit from earlier and more accurate diagnosis based on molecular biomarkers. Variations in individual SNPs have relatively small effects, but when several polymorphisms are combined in a complex pathway like *N*-glycosylation, the final product of the pathway - in this case IgG N-glycan - can be significantly different, with consequences for IgG function and possibly also disease susceptibility. Our results may also provide an explanation for the reported pleiotropy and antagonistic genetic effects of loci involved in autoimmune diseases and hematologic cancers [39], [77].

## Materials and Methods

### Ethics statement

All research in this study that involved human participants has been approved by the appropriate ethics committees: the Ethics Committee of the University of Split Medical School for all Croatian examinees from Vis and Korcula islands; the Local Research Ethics Committees in Orkney and Aberdeen for the Orkney Complex Disease Study (ORCADES); the University of Uppsala (Dnr 2005:325) for all examinees from Northern Sweden; the Leiden University Medical Centre Ethical Committee for all participants in the Leiden Longevity Study (LLS); and the Ethics Committee of the London School of Hygiene and Tropical Medicine for all SLE cases and controls from Trinidad. All ethics approvals were given in compliance with the Declaration of Helsinki (World Medical Association, 2000). All human subjects included in this study have signed appropriate informed consent.

### Study participants—discovery and replication cohorts

All population studies recruited adult individuals within a community irrespective of any specific phenotype. Fasting blood samples were collected, biochemical and physiological measurements taken and questionnaire data for medical history as well as lifestyle and environmental exposures were collected following similar protocols. Basic cohort descriptives are included in Table S11.

The CROATIA-Vis study includes 1008 Croatians, aged 18–93 years, who were recruited from the villages of Vis and Komiža on the Dalmatian island of Vis during 2003 and 2004 within a larger genetic epidemiology program [82]. The CROATIA-Korcula study includes 969 Croatians between the ages of 18 and 98 [83]. The field work was performed in 2007 and 2008 in the eastern part of the island, targeting healthy volunteers from the town of Korčula and the villages of Lumbarda, Žrnovo and Račišće.

The Orkney Complex Disease Study (ORCADES) was performed in the Scottish archipelago of Orkney and collected data between 2005 and 2011 [84]. Data for 889 participants aged 18 to 100 years from a subgroup of ten islands, were used for this analysis.

The Northern Swedish Population Health Study (NSPHS) is a family-based population study including a comprehensive health investigation and collection of data on family structure, lifestyle, diet, medical history and samples for laboratory analyses from peoples living in the north of Sweden [84]. Complete data were available from 179 participants aged 14 to 91 years.

DNA samples were genotyped according to the manufacturer's instructions on Illumina Infinium SNP bead microarrays (HumanHap300v1 for CROATIA-Vis, HumanHap300v2 for ORCADES and NSPHS and HumanCNV370v1 for CROATIA-Korcula). Genotypes were determined using Illumina BeadStudio software. Genotyping was successfully completed on 991 individuals from CROATIA-Vis, 953 from CROATIA-Korcula, 889 from ORCADES and 700 from NSPHS, providing a platform for genome-wide association study of multiple quantitative traits in these founder populations.

The Leiden Longevity Study (LLS) has been described in detail previously [85]. It is a family based study and consists of 1671 offspring of 421 nonagenarian sibling pairs of Dutch descent, and their 744 partners. 1848 individuals with available genotypic and IgG measurements data were included in the current analysis. Within the Leiden Longevity Study 1345 individuals were genotyped using Illumina660 W (Rotterdam, Netherlands) and 503 individuals were genotyped using Illumina OmniExpress (Estonian Biocentre, Genotyping Core Facility, Estonia).

### Isolation of IgG and glycan analysis

In the discovery population cohorts (CROATIA-Vis, CROATIA-Korcula, ORCADES, and NSPHS), the IgG was isolated using protein G plates and its glycans analysed by UPLC in 2247 individuals, as reported previously [19]. Briefly, IgG glycans were labelled with 2-AB fluorescent dye and separated by hydrophilic interaction ultra-performance liquid chromatography (UPLC). Glycans were separated into 24 chromatographic peaks and quantified as relative contributions of individual peaks to the total IgG glycome. The majority of peaks contained individual glycan structures, while some contained more structures. Relative intensities of each glycan structure in each UPLC peak were determined by mass spectrometry as reported previously [19]. On the basis of these 24 directly measured “glycan traits”, additional 54 “derived traits” were calculated. These include the percentage of galactosylation, fucosylation, sialylation, etc. described in the Table S1. When UPLC peaks containing multiple traits were used to calculate derived traits, only glycans with major contribution to fluorescence intensity were used.

In the replication population cohort (Leiden Longevity Study), the IgG was isolated from plasma samples of 1848 participants. Glycosylation patterns of IgG1 and IgG2 were investigated by analysis of tryptic glycopeptides using MALDI-TOF MS. Six glycoforms per IgG subclass were determined by MALDI-TOFMS. Since the intensities of all glycoforms were related to the monogalactosylated, core-fucosylated biantennary species (glycoform B), five relative intensities were registered per IgG subclass [86].

### Genotype and phenotype quality control

Genotyping quality control was performed using the same procedures for all four discovery populations (CROATIA-Vis, CROATIA-Korcula, ORCADES, and NSPHS). Individuals with a call rate less than 97% were removed as well as SNPs with a call rate less than 98% (95% for CROATIA-Vis), minor allele frequency less than 0.02 or Hardy-Weinberg equilibrium p-value less than 1×10^−10^. 924 individuals passed all quality control thresholds from CROATIA-Vis, 898 from CROATIA-Korcula, 889 from ORCADES and 656 from NSPHS.

Extreme outliers (those with values more than 3 times the interquartile distances away from either the 75th or the 25th percentile values) were removed for each glycan measure to account for errors in quantification and to remove individuals not representative of normal variation within the population. After phenotype quality control the number of individuals with complete phenotype and covariate information for the meta-analysis was 2247, consisting of 906 men and 1341 women (802 from CROATIA-Vis, 851 from CROATIA-Korcula, 415 from ORCADES, 179 from NSPHS).

In Leiden Longevity Study, GenomeStudio was used for genotyping calling algorithm. Sample call rate was >95%, and SNP exclusions criteria were Hardy-Weinberg equilibrium p value<10^−4^, SNP call rate<95%, and minor allele frequency <1%. The number of the overlapping SNPs that passed quality controls in both samples was 296,619.

To combine the data from the different array sets and to increase the overall coverage of the genome to up to 2.5 million SNPs, we imputed autosomal SNPs reported in the Haplotype Mapping Project (release #22, http://hapmap.ncbi.nlm.nih.gov) CEU sample. Based on the SNPs that were genotyped in all arrays and passed quality control, the imputation programmes MACH (http://www.sph.umich.edu/csg/abecasis/MACH/) or IMPUTE2 (http://mathgen.stats.ox.ac.uk/impute/impute_v2.html) were used to obtain ca. 2.5 million SNPs for further analysis.

For replication of genome-wide significant hits identified in the discovery meta-analysis, all SNPs listed in were used and looked up in LLS. The only exception was rs11621121, which had low imputation accuracy and did not pass quality control criteria. For this SNP, a set of 11 proxy SNPs from HapMap r. 22 (all with R^2^>0.85) was studied. All studied SNPs had imputation quality of 0.3 or greater.

### Genome-wide association analysis

In the discovery populations, genome-wide association analysis was firstly performed for each population and then combined using an inverse-variance weighted meta-analysis for all traits. Each trait was adjusted for sex, age and the first 3 principal components obtained from the population-specific identity-by-state (IBS) derived distances matrix. The residuals were transformed to ensure their normal distribution using quantile normalisation. Sex-specific analyses were adjusted for age and principal components only. The residuals expressed as z-scores were used for association analysis. The “mmscore” function of ProbABEL [87] was used for the association test under an additive model. This score test for family based association takes into account relationship structure and allowed unbiased estimations of SNP allelic effect when relatedness is present between examinees. The relationship matrix used in this analysis was generated by the “ibs” function of GenABEL (using weight = “freq” option), which uses genomic data to estimate the realized pair-wise kinship coefficient. All lambda values for the population-specific analyses were below 1.05 (Table S4), showing that this method efficiently accounts for family structure.

Inverse-variance weighted meta-analysis was performed using the MetABEL package (http://www.genabel.org) for R. SNPs with poor imputation quality (R^2^<0.3) were excluded prior to meta-analysis. Principal component analysis was performed using R to determine the number of independent traits used for these analyses (Table S10). 21 principal components explained 99% of the variance so an association was considered statistically significant at the genome-wide level if the p-value for an individual SNP was less than 2.27×10^−9^ (5×10^−8^/22 traits) [88]. SNPs were considered strongly suggestive with p-values between 5×10^−8^ and 2.27×10^−9^. Regions of association were visualized using the web-based software LocusZoom [89] to display the linkage disequilibrium (LD) of the region based on hg18/1000 Genomes June 1010 CEU data. The effect of the most significant SNP in each gene region expressed as percentage of the variance explained was calculated for each glycan trait adjusted for sex, age and first 3 principal components in each cohort individually using the “polygenic” function of the GenABEL package for R. Conditional analysis was undertaken for all significant and suggestive regions. GWAS was performed as described above with the additional adjustment for the dosage of the top SNP in the region for only the chromosome containing the association. Subsequent meta-analysis was performed as described previously and the results visualised using LocusZoom to ensure that the association peak have been removed.

In LLS, all IgG measurements were log-transformed. The score statistic for testing for an additive effect of a diallelic locus on quantitative phenotype was used. To account for relatedness in offspring data we used the kinship coefficients matrix when computing the variance of the score statistic. Imputation was dealt with by accounting for loss of information due to genotype uncertainty [90]. For the association analysis of the GWAS data, we applied the score test for the quantitative trait correcting for sex and age using an executable C++ program QTassoc (http://www.lumc.nl/uh, under GWAS Software). For further details we refer to supplementary online information.

### Experiments in Ikzf1 knockout mice

The *Ikzf1^+/−^* mice harbouring the Neo-PAX5-IRES-GFP knock in allele were obtained from Meinrad Busslinger (IMP, Vienna) and backcrossed to C57BL/6 mice. Both wild-type and *Ikzf1*Neo^+/−^ animals at the age of about 8 months were subjected to retro-orbital puncture to collect blood in the presence of EDTA. Samples were centrifuged for 10 minutes at room temperature and plasma was harvested. IgG was isolated and subjected to glycan analyses.

Statistical significance of the difference in distributions of IgG glycome between wild type and the *Ikzf1^+/−^* mice was assessed using empirical version of the Hotelling's test. In brief, the empirical distribution of the Hotelling's T^2^ statistics was worked out by permuting the group status of the animals at random without replacement 10,000 times. This empirical distribution was then contrasted with the original value of T^2^, with the proportion of empirically observed T^2^ values greater than or equal to the original T^2^ regarded as the empirical p-value.

### Dataset with SLE cases and matched controls

A total of 101 SLE cases and 183 controls from Trinidad were studied. The inclusion criteria for cases and controls in Trinidad were designed to restrict the sample to individuals without Indian or Chinese ancestry. Cases and controls were eligible to be included if they were resident in northern Trinidad (excluding the southern part of the island where Indians are in the majority) and they had Christian (rather than Hindu, Muslim or Chinese) first names. Identification of cases was carried out by contacting all physicians specializing in rheumatology, nephrology and dermatology at the two main public hospitals in northern Trinidad and asking for a list of all SLE patients from their out-patient clinics. At the main dermatology clinic a register of cases since 1992 was available. Furthermore, a systematic search of: (a) outpatient records at the two hospitals, (b) hospital laboratory test results positive for auto-antibodies (anti-nuclear or anti-double-stranded DNA antibody titre >1∶256) and (c) histological reports of skin biopsy examination consistent with SLE was performed. Lastly, SLE cases were also identified through the Lupus Society of Trinidad and Tobago (90% of those patients were also identified through one of the two main public hospitals). For each case, randomly chosen households in the same neighbourhood were sampled by the field team to obtain (where possible) two controls, matched with the case for sex and for 20-year age group. Cases and controls were interviewed at home or in the project office by using a custom made questionnaire.

The case definition of SLE was based on American Rheumatism Association (ARA) criteria [91], applied to medical records (available for more than 90% of cases), and to the medical history given by the patient. Informed consent for blood sampling and the use of the sample for genetic studies including estimation of admixture was obtained from each participant. Initial case ascertainment identified 264 possible cases of SLE. Of these, 72 (27%) were excluded either on the basis of their names or because their medical history did not meet ARA criteria for the diagnosis of SLE. Of the remaining 192 individuals, 54 had incomplete addresses or were not resident in northern Trinidad, four were too ill to be interviewed, eight were aged less than 18 years and two refused to participate. For 80% (99/124) of cases, two matched controls were obtained: the response rate from those invited to participate as controls was 70%. The total sample consisted of 124 cases and 219 controls aged over 20 years who completed the questionnaire. Blood samples were obtained from 122 cases and 219 controls and DNA was successfully extracted from 93% (317/341) of these. IgG glycans were successfully measured in 303 individuals. Age at sampling was not available for 17 individuals and 2 individuals were lost due to the ID mismatch.

To test predictive power of selected glycan trait, we fitted logistic regression models (including and excluding the glycan) and used predRisk function of PredictABEL package for R to evaluate the predictive ability.

## Supporting Information

Figure S1 Forrest plots for associations of glycan traits measured by UPLC and genetic polymorphisms.
(PPT)

Table S1 The description of 23 quantitative IgG glycosylation traits measured by UPLC and 54 derived traits.
(XLS)

Table S2 Descriptive statistics of glycan traits in discovery cohorts.
(XLS)

Table S3 Summary data for all single-nucleotide polymorphisms and traits with suggestive associations (p<1×10^-5^) with glycans measured by UPLC.
(XLS)

Table S4 Population-specific and pooled genomic control (GC) factors for associations with UPLC glycan traits.
(XLS)

Table S5 Description of five glycan traits measured by MS and their descriptive statistic in the replication cohort.
(XLS)

Table S6 Summary data for all single-nucleotide polymorphisms with replicated in the LLS cohort.
(XLS)

Table S7 IgG glycans in 5 heterozygous *Ikzf1* knockout mice and 5 wild-type controls.
(XLS)

Table S8 Data for all IgG N-glycans measured in 101 Afro-Caribbean cases with SLE and 183 controls (Extended Table 4 from the main manuscript).
(XLS)

Table S9 Effects of corticosteroids on IgG glycans.
(XLS)

Table S10 Principal component analysis of IgG glycosylation traits.
(XLS)

Table S11 Description of the analysed populations.
(XLS)
