# Supplementary material for: Loci Associated with N-Glycosylation of Human Immunoglobulin G Show Pleiotropy with Autoimmune Diseases and Haematological Cancers
Source: PLoS Genet. 2013 Jan 31;9(1):e1003225. doi: 10.1371/journal.pgen.1003225 (PMC3561084; doi:10.1371/journal.pgen.1003225)
Supplement: Figure S1 — Forrest plots for associations of glycan traits measured by UPLC and genetic polymorphisms. (PPT) [file pgen.1003225.s001.ppt]

## Slide 1
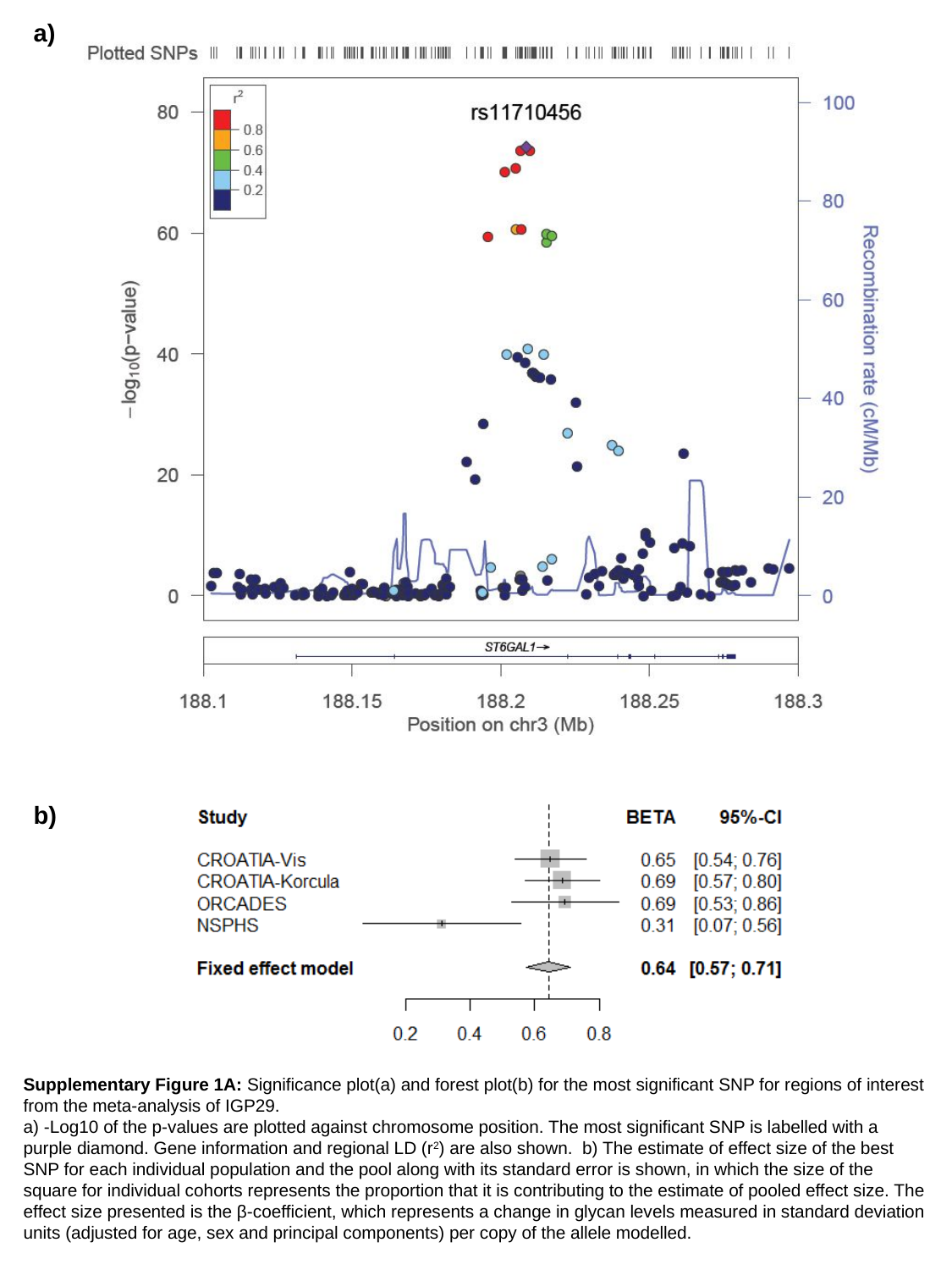

a)
b)
Supplementary Figure 1A: Significance plot(a) and forest plot(b) for the most significant SNP for regions of interest from the meta-analysis of IGP29.
a) -Log10 of the p-values are plotted against chromosome position. The most significant SNP is labelled with a purple diamond. Gene information and regional LD (r2) are also shown. b) The estimate of effect size of the best SNP for each individual population and the pool along with its standard error is shown, in which the size of the square for individual cohorts represents the proportion that it is contributing to the estimate of pooled effect size. The effect size presented is the β-coefficient, which represents a change in glycan levels measured in standard deviation units (adjusted for age, sex and principal components) per copy of the allele modelled.

## Slide 2
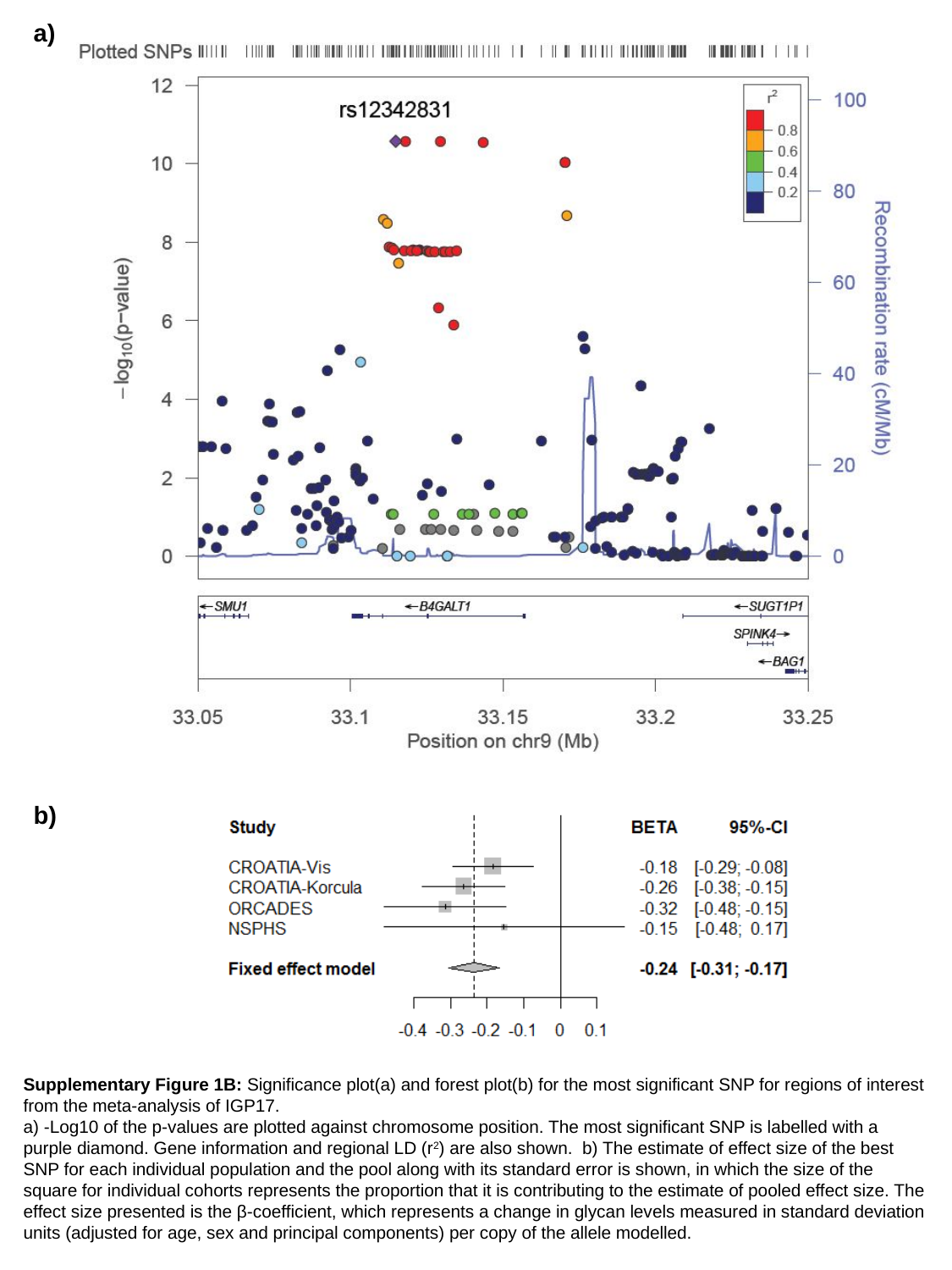

a)
b)
Supplementary Figure 1B: Significance plot(a) and forest plot(b) for the most significant SNP for regions of interest from the meta-analysis of IGP17.
a) -Log10 of the p-values are plotted against chromosome position. The most significant SNP is labelled with a purple diamond. Gene information and regional LD (r2) are also shown. b) The estimate of effect size of the best SNP for each individual population and the pool along with its standard error is shown, in which the size of the square for individual cohorts represents the proportion that it is contributing to the estimate of pooled effect size. The effect size presented is the β-coefficient, which represents a change in glycan levels measured in standard deviation units (adjusted for age, sex and principal components) per copy of the allele modelled.

## Slide 3
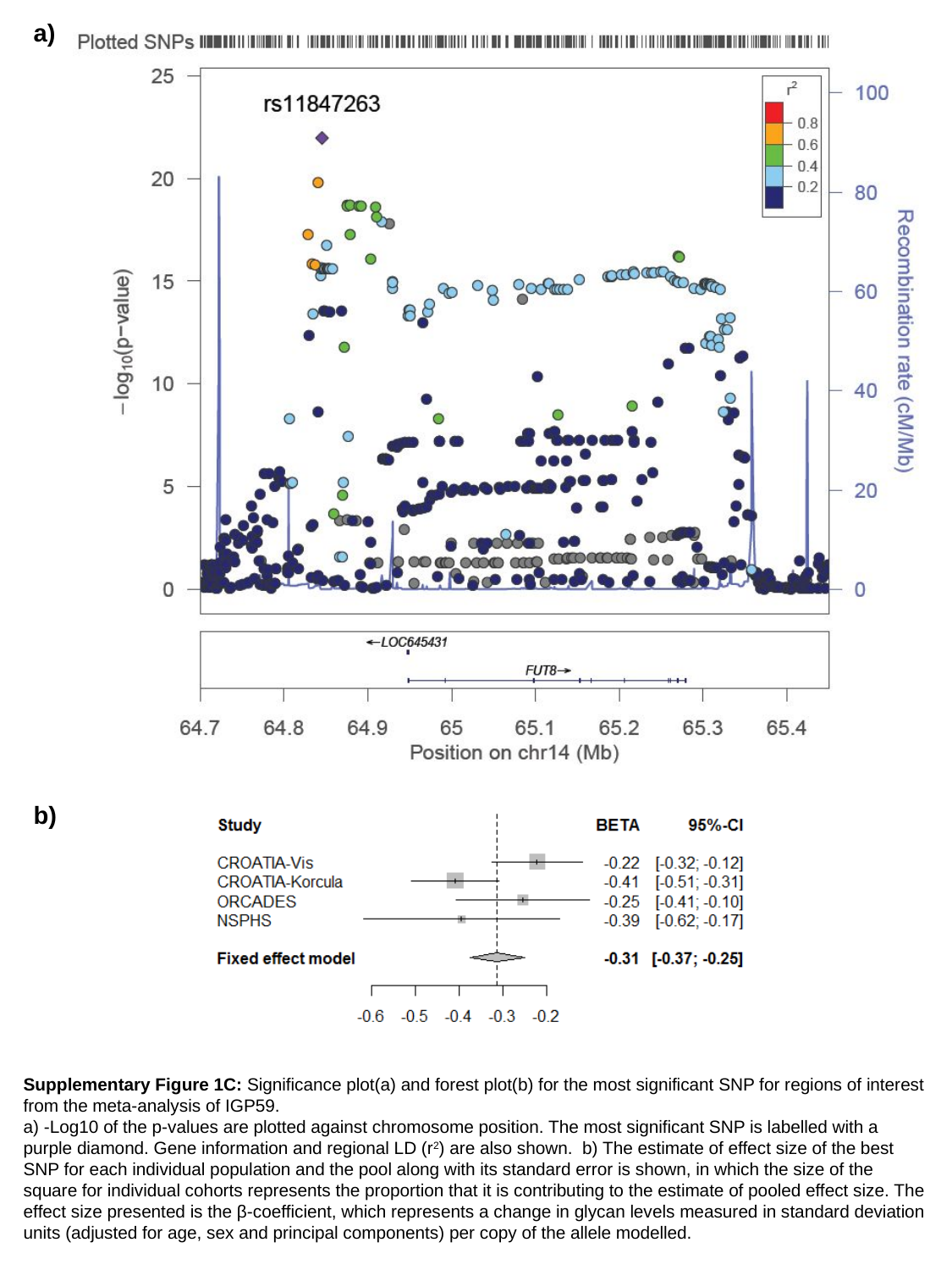

a)
b)
Supplementary Figure 1C: Significance plot(a) and forest plot(b) for the most significant SNP for regions of interest from the meta-analysis of IGP59.
a) -Log10 of the p-values are plotted against chromosome position. The most significant SNP is labelled with a purple diamond. Gene information and regional LD (r2) are also shown. b) The estimate of effect size of the best SNP for each individual population and the pool along with its standard error is shown, in which the size of the square for individual cohorts represents the proportion that it is contributing to the estimate of pooled effect size. The effect size presented is the β-coefficient, which represents a change in glycan levels measured in standard deviation units (adjusted for age, sex and principal components) per copy of the allele modelled.

## Slide 4
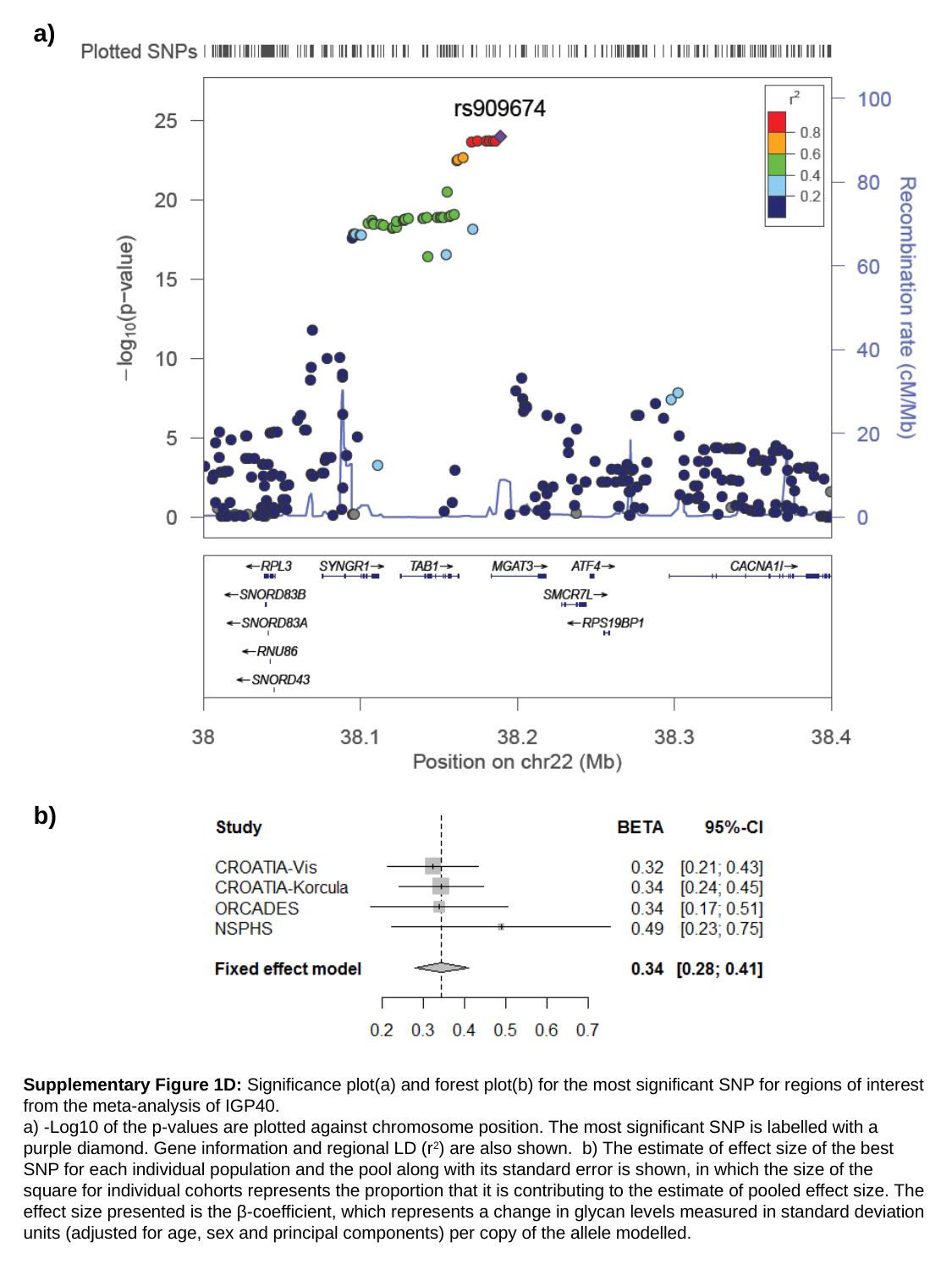

a)
b)
Supplementary Figure 1D: Significance plot(a) and forest plot(b) for the most significant SNP for regions of interest from the meta-analysis of IGP40.
a) -Log10 of the p-values are plotted against chromosome position. The most significant SNP is labelled with a purple diamond. Gene information and regional LD (r2) are also shown. b) The estimate of effect size of the best SNP for each individual population and the pool along with its standard error is shown, in which the size of the square for individual cohorts represents the proportion that it is contributing to the estimate of pooled effect size. The effect size presented is the β-coefficient, which represents a change in glycan levels measured in standard deviation units (adjusted for age, sex and principal components) per copy of the allele modelled.

## Slide 5
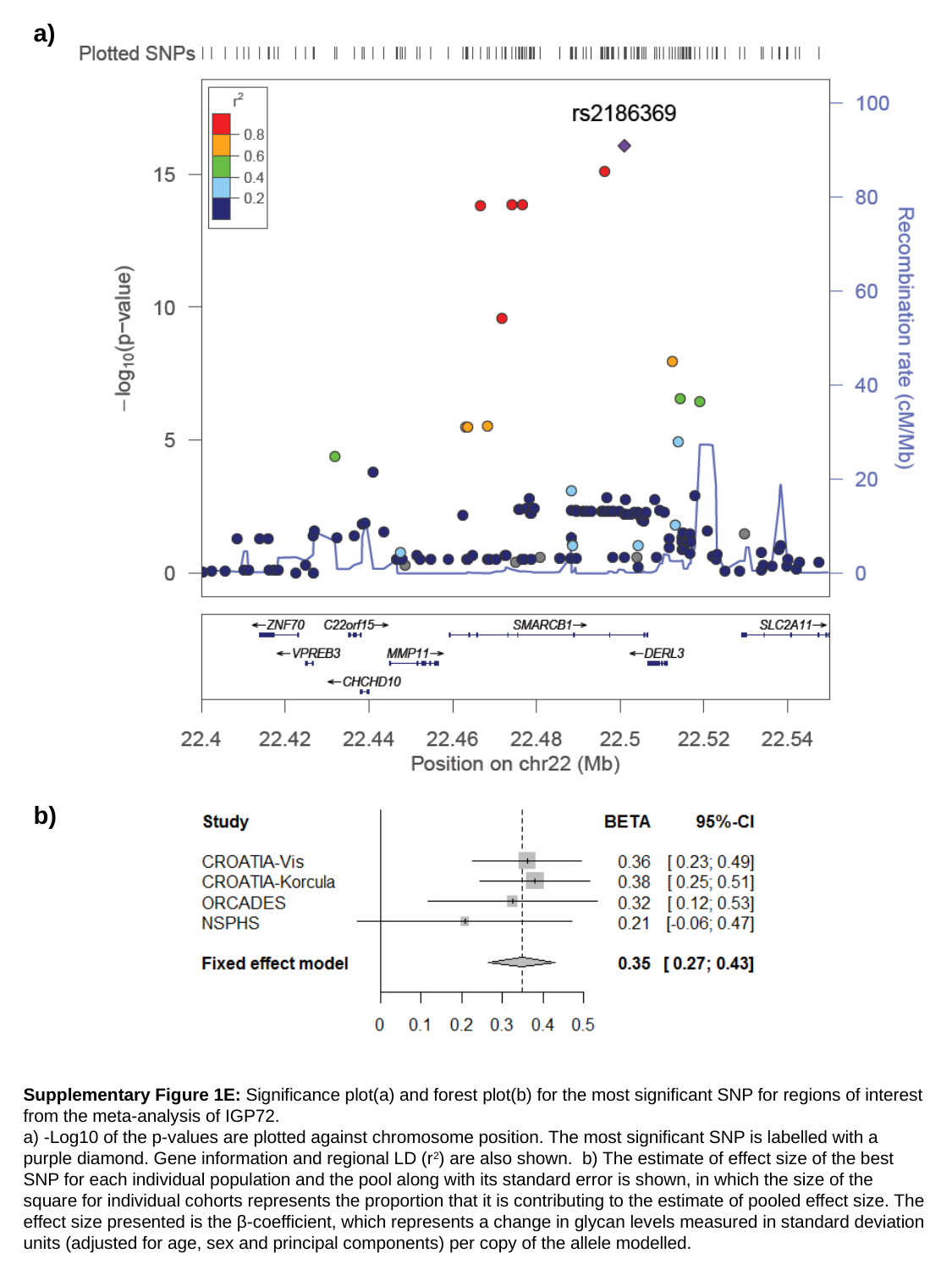

a)
b)
Supplementary Figure 1E: Significance plot(a) and forest plot(b) for the most significant SNP for regions of interest from the meta-analysis of IGP72.
a) -Log10 of the p-values are plotted against chromosome position. The most significant SNP is labelled with a purple diamond. Gene information and regional LD (r2) are also shown. b) The estimate of effect size of the best SNP for each individual population and the pool along with its standard error is shown, in which the size of the square for individual cohorts represents the proportion that it is contributing to the estimate of pooled effect size. The effect size presented is the β-coefficient, which represents a change in glycan levels measured in standard deviation units (adjusted for age, sex and principal components) per copy of the allele modelled.

## Slide 6
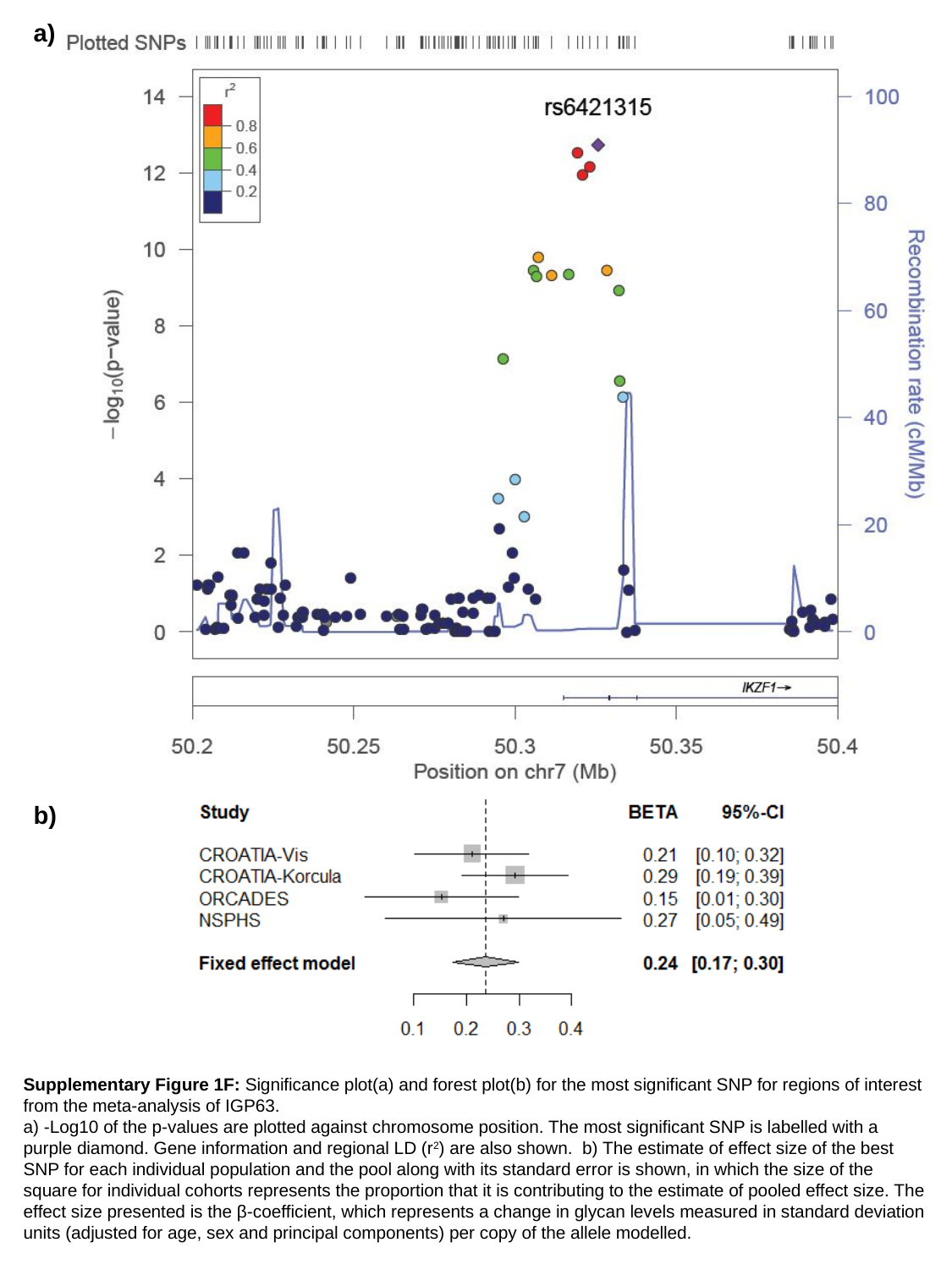

a)
b)
Supplementary Figure 1F: Significance plot(a) and forest plot(b) for the most significant SNP for regions of interest from the meta-analysis of IGP63.
a) -Log10 of the p-values are plotted against chromosome position. The most significant SNP is labelled with a purple diamond. Gene information and regional LD (r2) are also shown. b) The estimate of effect size of the best SNP for each individual population and the pool along with its standard error is shown, in which the size of the square for individual cohorts represents the proportion that it is contributing to the estimate of pooled effect size. The effect size presented is the β-coefficient, which represents a change in glycan levels measured in standard deviation units (adjusted for age, sex and principal components) per copy of the allele modelled.

## Slide 7
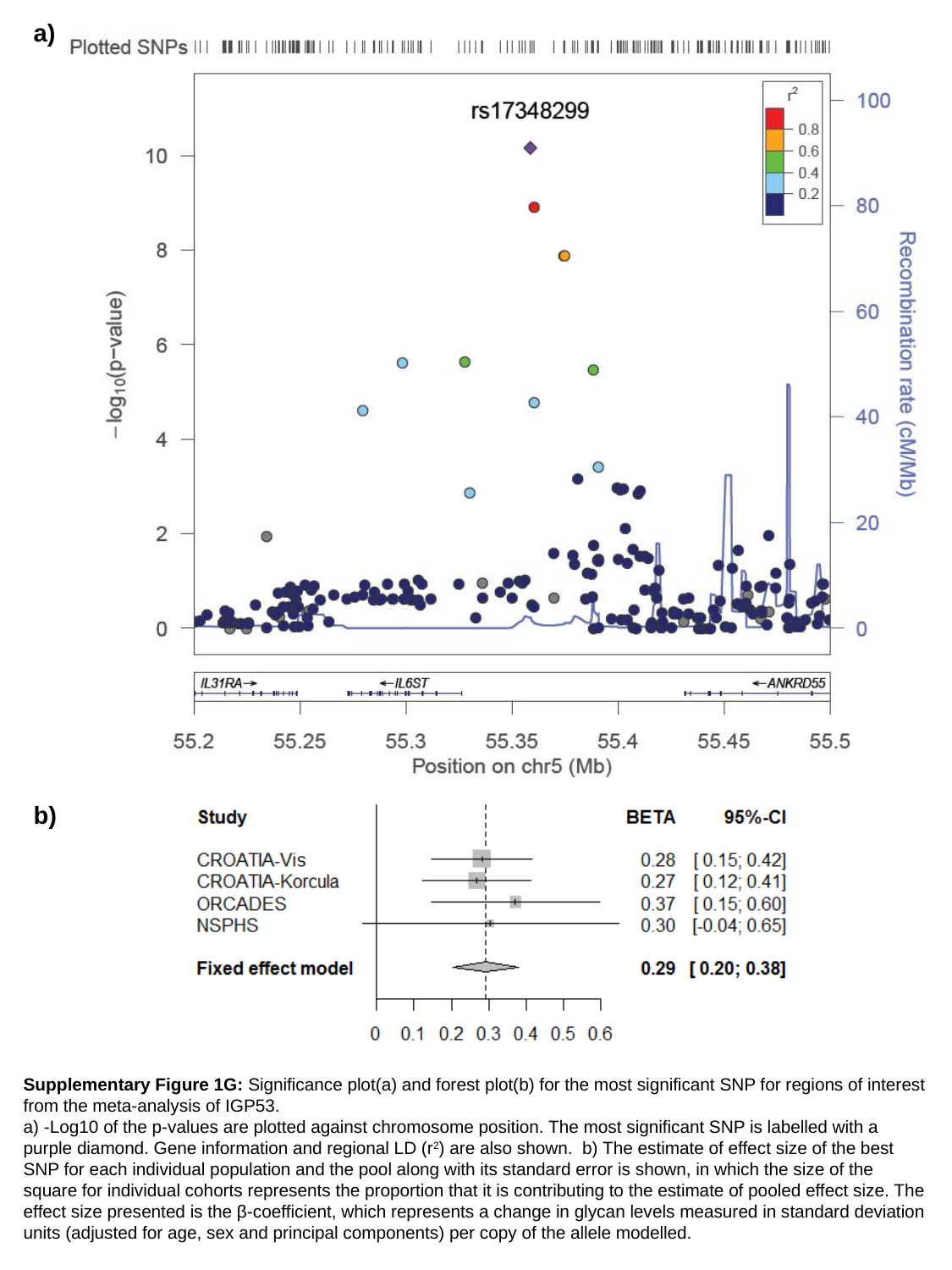

a)
b)
Supplementary Figure 1G: Significance plot(a) and forest plot(b) for the most significant SNP for regions of interest from the meta-analysis of IGP53.
a) -Log10 of the p-values are plotted against chromosome position. The most significant SNP is labelled with a purple diamond. Gene information and regional LD (r2) are also shown. b) The estimate of effect size of the best SNP for each individual population and the pool along with its standard error is shown, in which the size of the square for individual cohorts represents the proportion that it is contributing to the estimate of pooled effect size. The effect size presented is the β-coefficient, which represents a change in glycan levels measured in standard deviation units (adjusted for age, sex and principal components) per copy of the allele modelled.

## Slide 8
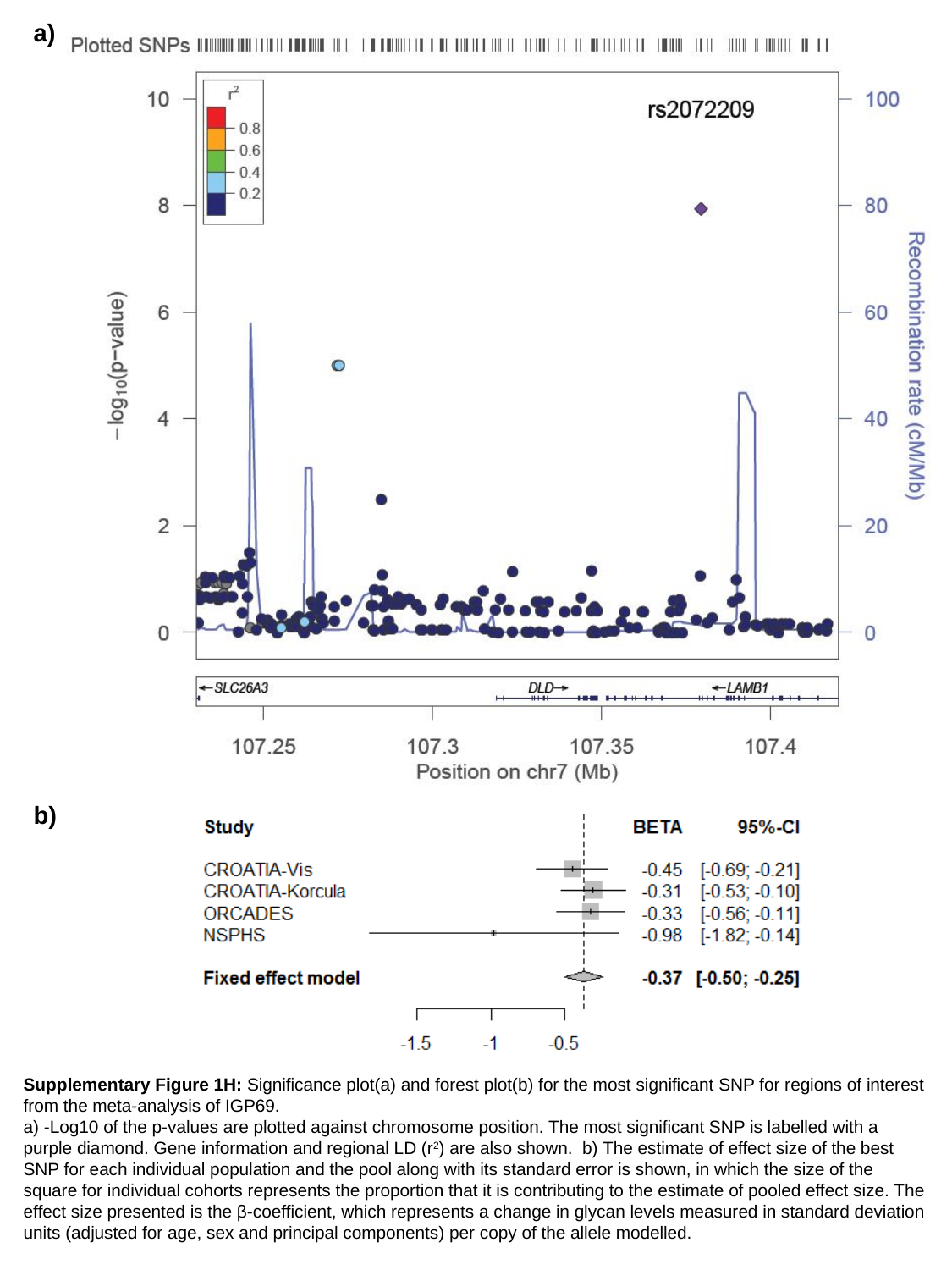

a)
b)
Supplementary Figure 1H: Significance plot(a) and forest plot(b) for the most significant SNP for regions of interest from the meta-analysis of IGP69.
a) -Log10 of the p-values are plotted against chromosome position. The most significant SNP is labelled with a purple diamond. Gene information and regional LD (r2) are also shown. b) The estimate of effect size of the best SNP for each individual population and the pool along with its standard error is shown, in which the size of the square for individual cohorts represents the proportion that it is contributing to the estimate of pooled effect size. The effect size presented is the β-coefficient, which represents a change in glycan levels measured in standard deviation units (adjusted for age, sex and principal components) per copy of the allele modelled.

## Slide 9
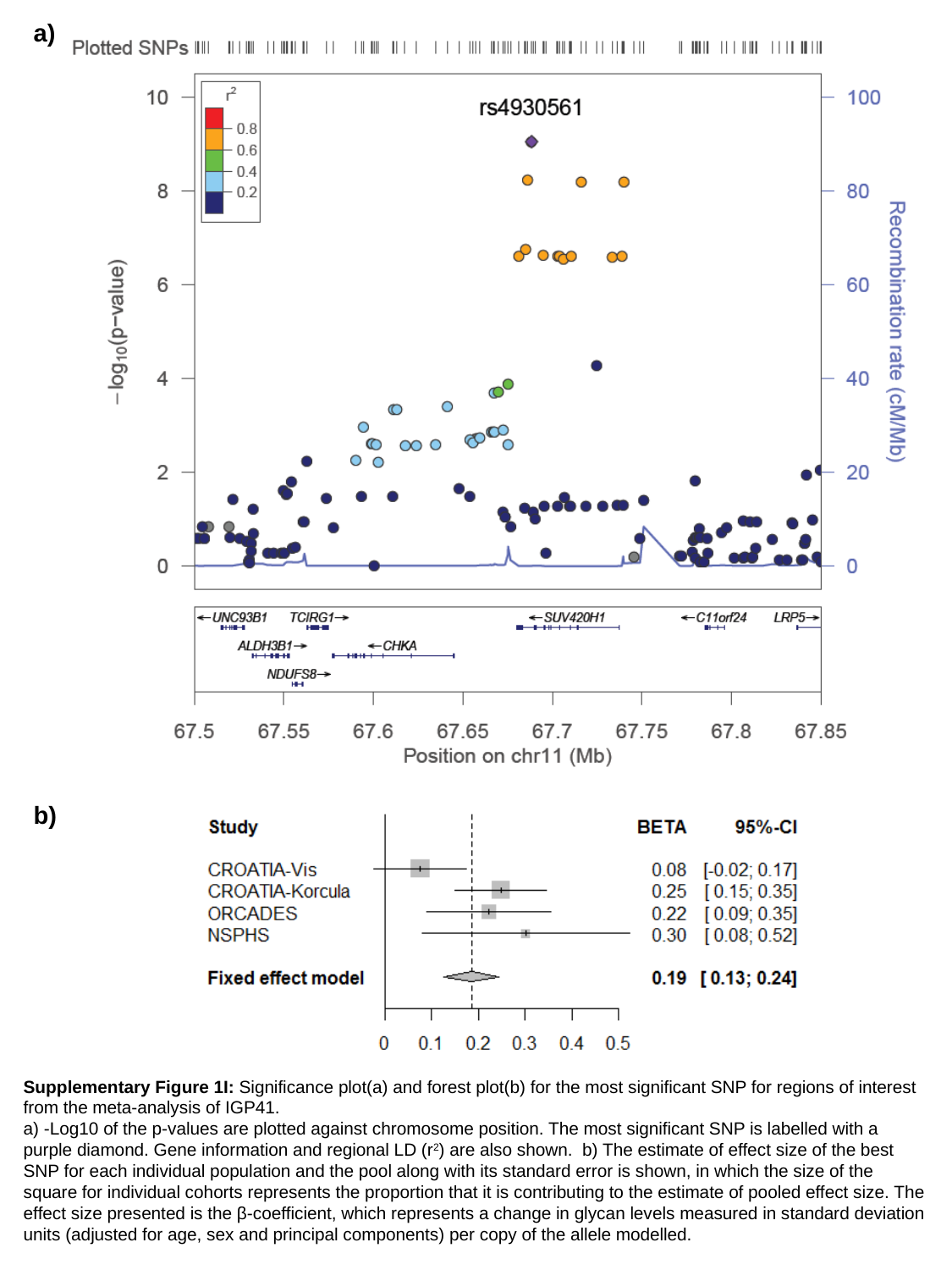

a)
b)
Supplementary Figure 1I: Significance plot(a) and forest plot(b) for the most significant SNP for regions of interest from the meta-analysis of IGP41.
a) -Log10 of the p-values are plotted against chromosome position. The most significant SNP is labelled with a purple diamond. Gene information and regional LD (r2) are also shown. b) The estimate of effect size of the best SNP for each individual population and the pool along with its standard error is shown, in which the size of the square for individual cohorts represents the proportion that it is contributing to the estimate of pooled effect size. The effect size presented is the β-coefficient, which represents a change in glycan levels measured in standard deviation units (adjusted for age, sex and principal components) per copy of the allele modelled.

## Slide 10
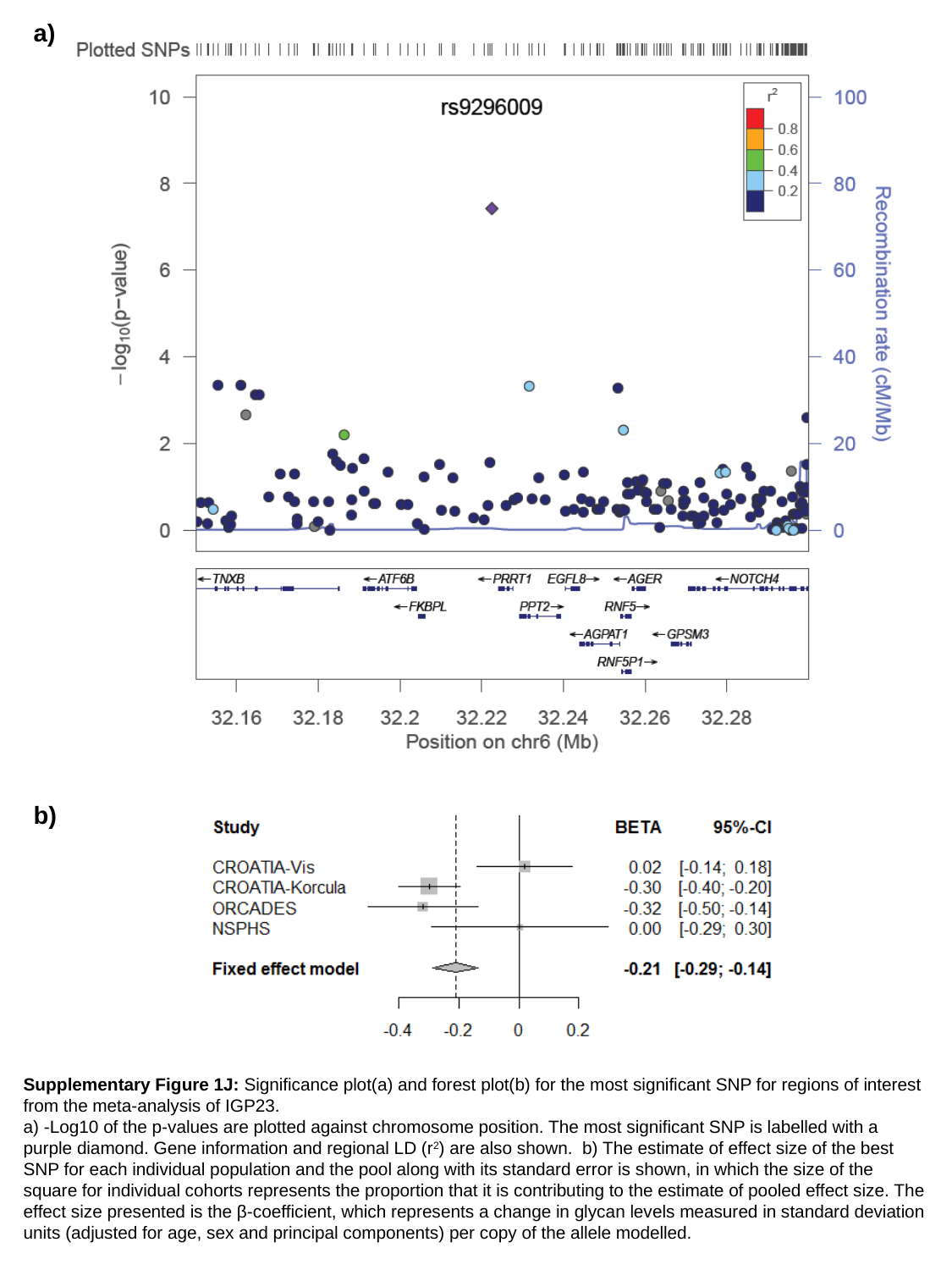

a)
b)
Supplementary Figure 1J: Significance plot(a) and forest plot(b) for the most significant SNP for regions of interest from the meta-analysis of IGP23.
a) -Log10 of the p-values are plotted against chromosome position. The most significant SNP is labelled with a purple diamond. Gene information and regional LD (r2) are also shown. b) The estimate of effect size of the best SNP for each individual population and the pool along with its standard error is shown, in which the size of the square for individual cohorts represents the proportion that it is contributing to the estimate of pooled effect size. The effect size presented is the β-coefficient, which represents a change in glycan levels measured in standard deviation units (adjusted for age, sex and principal components) per copy of the allele modelled.

## Slide 11
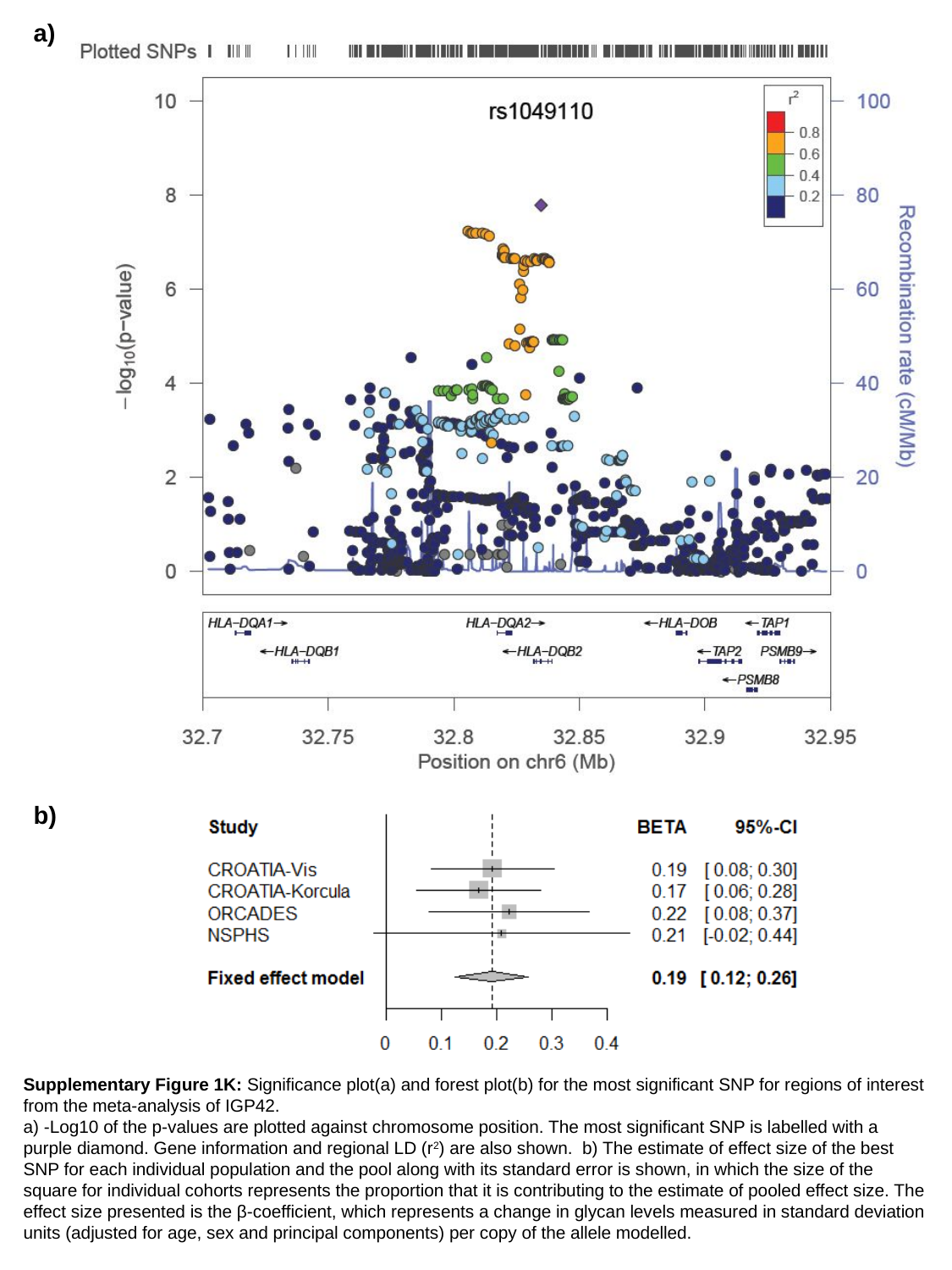

a)
b)
Supplementary Figure 1K: Significance plot(a) and forest plot(b) for the most significant SNP for regions of interest from the meta-analysis of IGP42.
a) -Log10 of the p-values are plotted against chromosome position. The most significant SNP is labelled with a purple diamond. Gene information and regional LD (r2) are also shown. b) The estimate of effect size of the best SNP for each individual population and the pool along with its standard error is shown, in which the size of the square for individual cohorts represents the proportion that it is contributing to the estimate of pooled effect size. The effect size presented is the β-coefficient, which represents a change in glycan levels measured in standard deviation units (adjusted for age, sex and principal components) per copy of the allele modelled.

## Slide 12
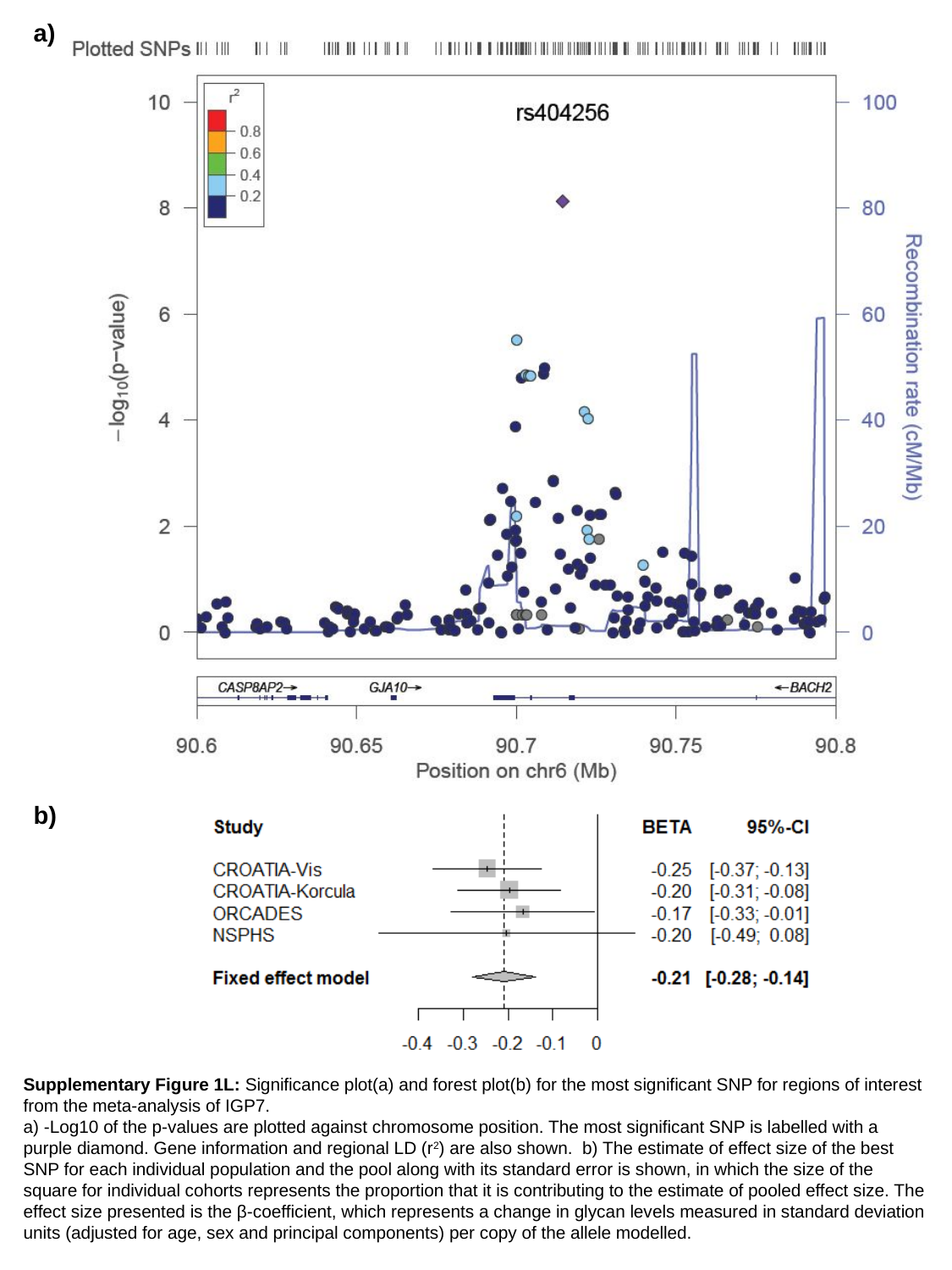

a)
b)
Supplementary Figure 1L: Significance plot(a) and forest plot(b) for the most significant SNP for regions of interest from the meta-analysis of IGP7.
a) -Log10 of the p-values are plotted against chromosome position. The most significant SNP is labelled with a purple diamond. Gene information and regional LD (r2) are also shown. b) The estimate of effect size of the best SNP for each individual population and the pool along with its standard error is shown, in which the size of the square for individual cohorts represents the proportion that it is contributing to the estimate of pooled effect size. The effect size presented is the β-coefficient, which represents a change in glycan levels measured in standard deviation units (adjusted for age, sex and principal components) per copy of the allele modelled.

## Slide 13
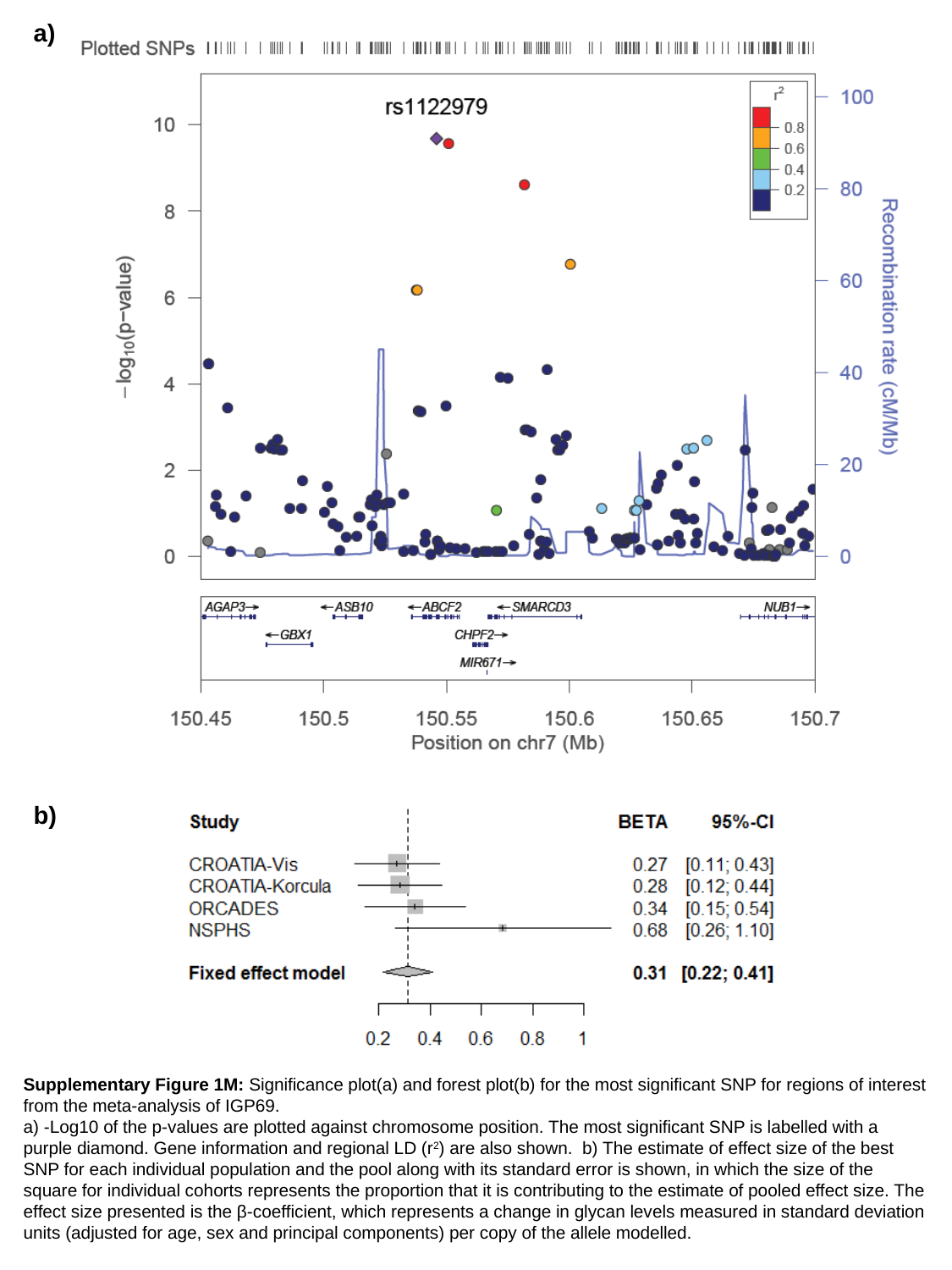

a)
b)
Supplementary Figure 1M: Significance plot(a) and forest plot(b) for the most significant SNP for regions of interest from the meta-analysis of IGP69.
a) -Log10 of the p-values are plotted against chromosome position. The most significant SNP is labelled with a purple diamond. Gene information and regional LD (r2) are also shown. b) The estimate of effect size of the best SNP for each individual population and the pool along with its standard error is shown, in which the size of the square for individual cohorts represents the proportion that it is contributing to the estimate of pooled effect size. The effect size presented is the β-coefficient, which represents a change in glycan levels measured in standard deviation units (adjusted for age, sex and principal components) per copy of the allele modelled.

## Slide 14
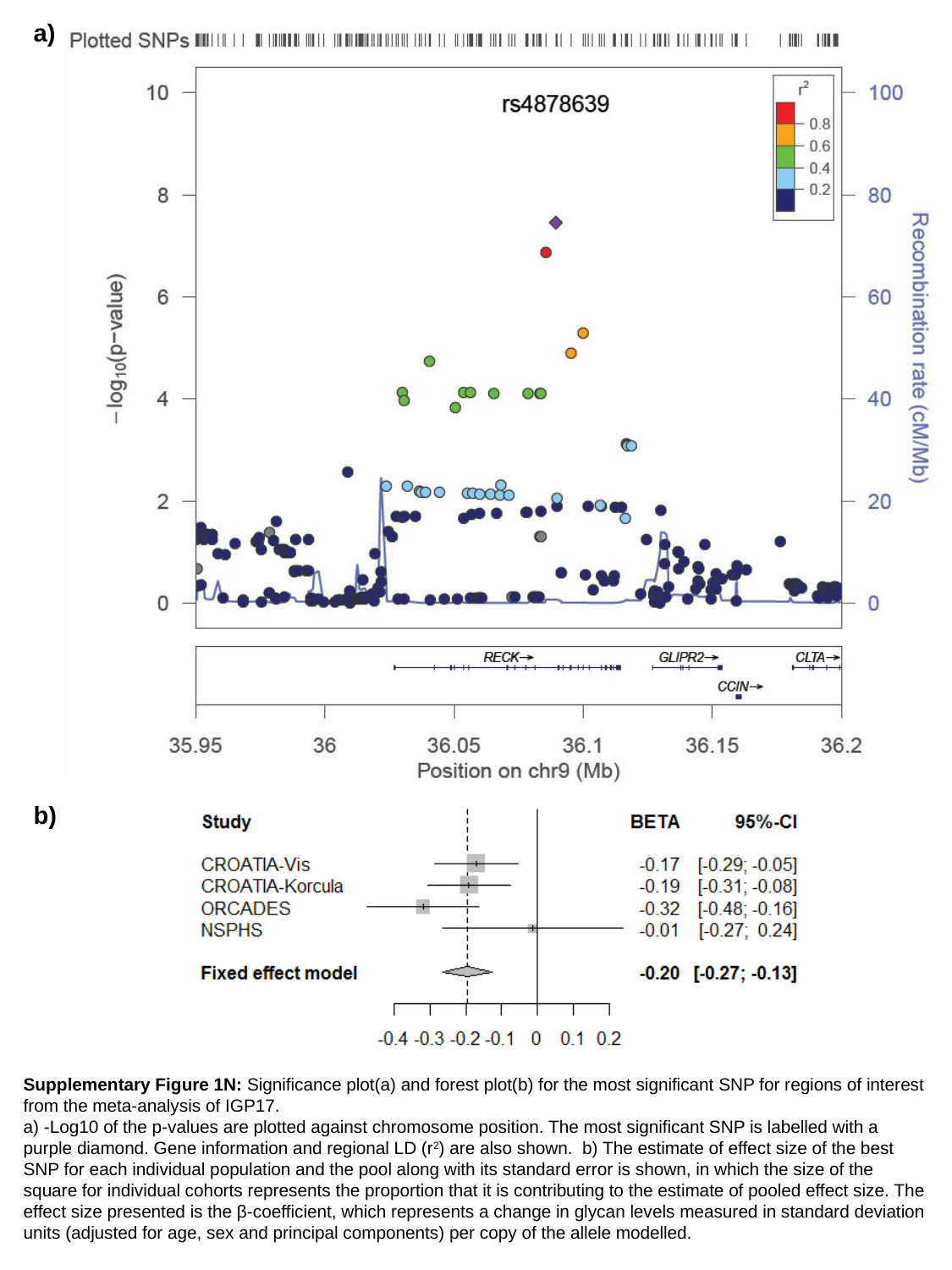

a)
b)
Supplementary Figure 1N: Significance plot(a) and forest plot(b) for the most significant SNP for regions of interest from the meta-analysis of IGP17.
a) -Log10 of the p-values are plotted against chromosome position. The most significant SNP is labelled with a purple diamond. Gene information and regional LD (r2) are also shown. b) The estimate of effect size of the best SNP for each individual population and the pool along with its standard error is shown, in which the size of the square for individual cohorts represents the proportion that it is contributing to the estimate of pooled effect size. The effect size presented is the β-coefficient, which represents a change in glycan levels measured in standard deviation units (adjusted for age, sex and principal components) per copy of the allele modelled.

## Slide 15
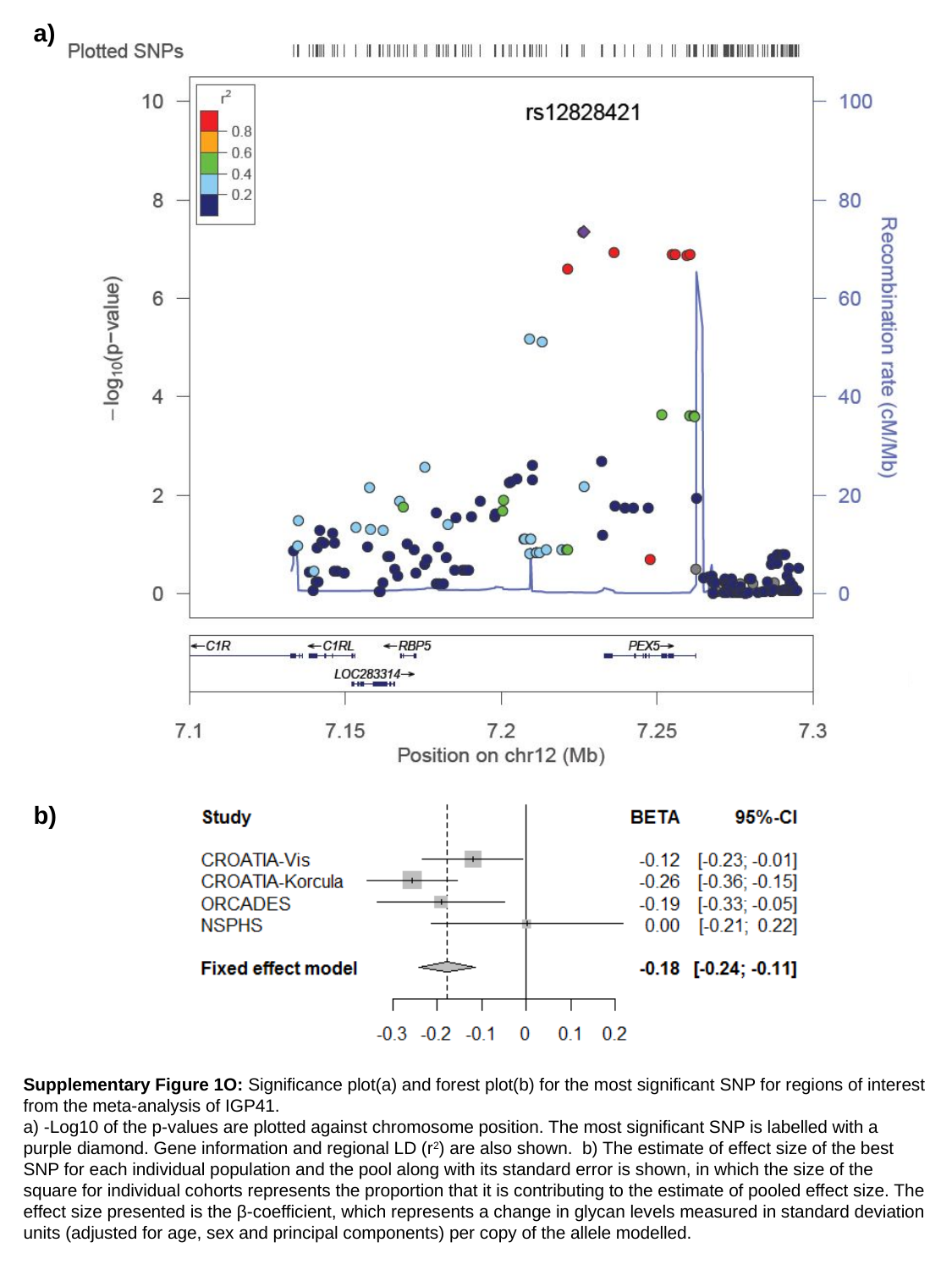

a)
b)
Supplementary Figure 1O: Significance plot(a) and forest plot(b) for the most significant SNP for regions of interest from the meta-analysis of IGP41.
a) -Log10 of the p-values are plotted against chromosome position. The most significant SNP is labelled with a purple diamond. Gene information and regional LD (r2) are also shown. b) The estimate of effect size of the best SNP for each individual population and the pool along with its standard error is shown, in which the size of the square for individual cohorts represents the proportion that it is contributing to the estimate of pooled effect size. The effect size presented is the β-coefficient, which represents a change in glycan levels measured in standard deviation units (adjusted for age, sex and principal components) per copy of the allele modelled.

## Slide 16
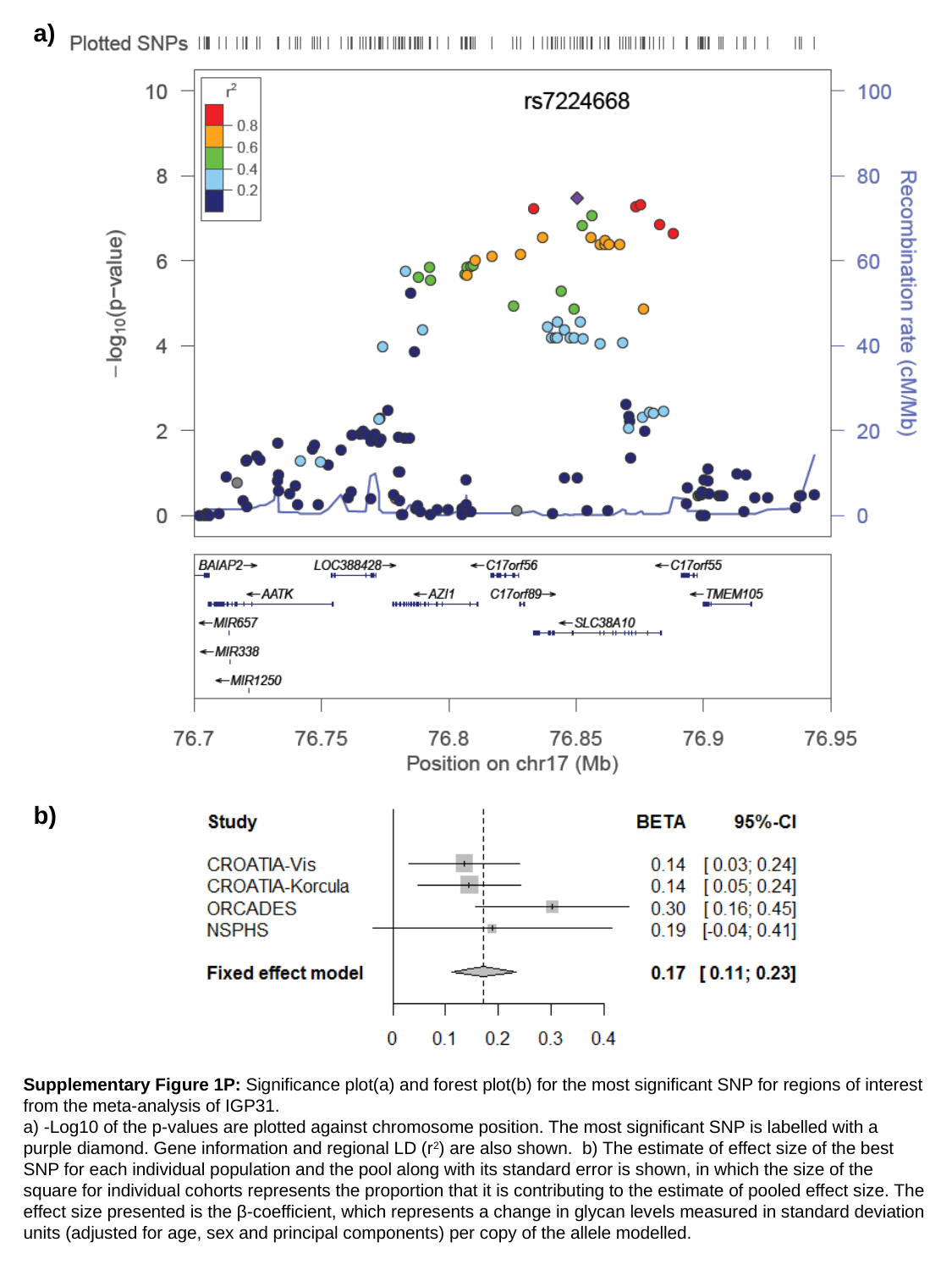

a)
b)
Supplementary Figure 1P: Significance plot(a) and forest plot(b) for the most significant SNP for regions of interest from the meta-analysis of IGP31.
a) -Log10 of the p-values are plotted against chromosome position. The most significant SNP is labelled with a purple diamond. Gene information and regional LD (r2) are also shown. b) The estimate of effect size of the best SNP for each individual population and the pool along with its standard error is shown, in which the size of the square for individual cohorts represents the proportion that it is contributing to the estimate of pooled effect size. The effect size presented is the β-coefficient, which represents a change in glycan levels measured in standard deviation units (adjusted for age, sex and principal components) per copy of the allele modelled.
